# Indicators and Instruments to Assess Components of Disability in Community-Dwelling Older Adults: A Systematic Review

**DOI:** 10.3390/s22218270

**Published:** 2022-10-28

**Authors:** Juliana Santos Moreira, Ana Melo, Rubim Santos, Andreia S. P. Sousa

**Affiliations:** 1 Center for Rehabilitation Research—Human Movement System (Re)habilitation Area, Department of Physiotherapy, School of Health, Polytechnic of Porto, 4200-072 Porto, Portugal; 2 Research Center in Physical Activity, Health and Leisure (CIAFEL), Faculty of Sport, University of Porto (FADEUP), 4200-450 Porto, Portugal; 3 Center for Rehabilitation Research—Human Movement System (Re)habilitation Area, Department of Physics, School of Health, Polytechnic of Porto, 4200-072 Porto, Portugal

**Keywords:** older adults, disability, indicators, aging, systematic review

## Abstract

The epidemiological demands of aging point to the need for characterizing older adults regarding health and disability. This systematic review aims to summarize the indicators (instruments) identifying different components of disability as a result of aging exposition in community-dwelling older adults, considering the International Classification of Functioning, Disability, and Health framework. Taking the PRISMA 2020 recommendations as a reference, studies with community-dwelling older adults, reporting the development and/or age disability modifications were included. Two reviewers analyzed the observational studies searched in the MEDLINE, CINAHL, Web of Science, Scopus, and Embase databases. Of the 137 potentially eligible studies, 49 were included in this review. Several indicators (instruments) demonstrated older adults’ disabilities according to the different domains of the ICF. Objective measures assessed Body Structures, Body Functions, and Environmental Factors and included handgrip strength (dynamometry, *n* = 8), cognitive function (Mini-Mental State examination, *n* = 7), gait speed (walk test, *n* = 6), and endurance (Chair stand-test, *n* = 4). Self-reported measures assessed Activities and Participation, but not the Body Structures, and included the basic and instrumental activities of daily living (ADL) (the Katz Index of ADL, *n* = 4 studies, the Lawton and Brody Instrumental ADL, *n* = 4 studies). The summary of the measures gathered can guide researchers and health professionals to select indicators (instruments) to assess and monitor older adults’ disabilities resulting from aging exposition, to support the development of new wearables, and to provide improvements to the existing ones, allowing the tailored assessment of different health and disability dimensions.

## 1. Introduction

Population aging, a consequence of long-term decreased fertility rates combined with increased life expectancy, has led to a contemporary global phenomenon responsible for a multisector societal transformation [1,2]. This transformation highlights the need to characterize the older adult population in terms of health, disability, and morbidity measures [2,3,4,5] to identify public health problems, plan and evaluate health policies, and guide public health interventions for older adults [6,7].

Comprehensively, aging is a physiological process modulated by several factors, namely, genetic, epigenetic, lifestyle, and environmental [8]. Through the influence of these modulators, individuals of the same chronological age present significant differences in health, disease, and disability [8,9]. It should also be considered that age represents a primary risk factor for chronic diseases, including cardiovascular, malignancy, and neurodegenerative conditions, which seem to strongly contribute to older adults’ disabilities [9].

The disability concept itself lacks a consensus definition, therefore, different diagnostic measures have been considered [10,11,12]. Nagi, 1965, proposed a theoretical model dividing the disability process into a pathway of four stages or components. The last stage, described as the limitations in activities of daily living (ADL), is characterized by a restriction in the performance of socially defined roles within a social–cultural and physical environment [11]. Later, Verbrugge and Jette, 1994, extended this model, theorizing that extra-individual factors (such as environmental), intra-individual, and risk factors, could influence the pathway that leads to disability [12]. Considering these models, the World Health Organization developed, in 2001, the International Classification of Functioning, Disability, and Health (ICF), which states that “disability serves as an umbrella term for impairments, activity limitations or participation restrictions”. The ICF organizes information into two categories, which are Functioning and Disability (Body Functions and Structures, Activities and Participation) and Contextual Factors (Environmental Factors, Personal Factors), but it is not an instrument for measuring disability [13]. Disability assessment has been carried out with self-reported information and/or performance-based measurements [9]. Despite the numerous disability measurements [14,15,16], the considerable heterogeneity in the way that disability was defined and categorized has been pointed out as a limitation in previous systematic reviews focused on risk factors [14], prognostic factors [15], and the prevalence of disability [16]. Moreover, different measures can result in diverse within-survey estimates of disability prevalence, unrelated health profiles of defined groups, and inequalities in the outcomes of people defined as having a disability [17]. Two systematic reviews began the process of defining disability measures for the older adult population [18,19]. One identified measures of mobility disability in community-dwelling older adults, outlining the need for standardizing instruments for comparison across studies, and for a better understanding of indicators and outcomes of mobility limitation in this population [18]. Yang et al., 2014, analyzed the contents and formats of general self-reported questionnaires on disability for older adults, gathering 24 questionnaires and comparing their content and formats, stating the difficulty in selecting the best one [19]. Despite the encouraging progress, there is still no definition of the measures that should be used considering all domains of the ICF and the use of both self-reported and objective measures to assess/monitor the influence of aging on older adults’ disabilities.

The measurement of the different components of disability according to the ICF framework has an essential role in understanding the aging process and its implications and in planning health programs and services. Moreover, the definition of indicators for different components of disability in older adults could also bring a significant contribution to the development of sensors to assess disability in all ICF domains. Accordingly, the purpose of this study is to systematically review indicators and respective instruments capable of identifying different components of disability as a result of aging exposition, in community-dwelling older adults, taking the ICF framework into consideration. Specifically, the ICF domains (Body Functions and Structures, Activities and Participation) and Contextual Factors (Environmental Factors) will be used as the reference.

## 2. Materials and Methods

The Preferred Reporting Items for Systematic Reviews and Meta-Analysis (PRISMA 2020) [20] was used as a reference. Systematic reviews should follow a protocol to reduce bias and ensure the findings are reproducible [21]; therefore, the present systematic review protocol was registered in PROSPERO, the International Prospective Register of Systematic Reviews, with the registration number CRD42021243416, and is available at https://www.crd.york.ac.uk/prospero/display_record.php?ID=CRD42021243416 (accessed on 15 August 2022).

### 2.1. Eligibility Criteria

The studies had to include community-dwelling older adults (60 years and over) and report the development and/or age modifications of the components of the disability measures according to the different ICF domains (Body structures, Body Functions, Activities and participation, and Environmental Factors). Observational studies, including cohort and cross-sectional designs, published in English, French, Spanish, or Portuguese, were included. Considering that the aim of the review is to gather the indicators and respective instruments to assess community older adults’ disability components, the exclusion criteria for the studies were: (1) studies that exclusively considered older adults’ frailty measures, or disability measures specifically designed to evaluate particular pathologies or populations; (2) studies that included individuals with a history of lesions with increasing disability-adjusted life-years, [22,23] and a high risk of long-term disability [24,25,26] in all domains of the ICF [27], as well as stroke and dementia, as these conditions are assessed by particular instruments focused on the involved pathological conditions; (3) studies including institutionalized older adults; and (4) studies where the design was meta-analysis, reviews, randomized and non-randomized controlled trials, case reports, pilot studies, technical notes, letters, editorials, or studies published as conference proceedings.

### 2.2. Information Sources and Search Strategy

A systematic search was performed in the electronic databases MEDLINE (PubMed), CINAHL, Web of Science, Scopus, and Embase on the 8 April 2021. Individual search strings were defined for every database, using medical subject headings (MeSH) and free text words in the title and abstract. The reference lists of selected studies, and relevant systematic analysis, were checked by two reviewers to identify the additional potential of eligible studies. The search strategy, for each database, can be accessed in the Supplementary Information.

### 2.3. Selection and Data Collection Process

Following the database search, all potentially eligible studies were retrieved and organized in the EndNote™ (Clarivate™, London, UK), version 20, software, where the duplicate publications were deleted. Studies’ titles and abstracts were independently assessed by two reviewers, who registered the reasons for exclusion in a table format. Disagreements between the two main reviewers were solved by consensus or by consulting a third independent reviewer in cases where consensus was not achieved. Additionally, two reviewers assessed the full texts of the potentially relevant studies. The flow of the search was displayed according to recommendations made by the PRISMA statement [20].

The data extraction of the included studies was performed by two reviewers using a pre-defined data extraction table, which retrieved the study identification, study design, sample characteristics, and the indicators with the respective instruments to evaluate them. Missing data from selected studies were requested from the authors via three emails sent over the course of a month and a half. 

### 2.4. Assessment of Methodologic Quality

The methodological quality risk of bias (RoB) assessment of selected studies is essential for interpreting the results, evaluating the strength of evidence found in any systematic review, and reporting information to guide and improve the quality of studies in a particular research area [21,28]. Despite the relevance of the RoB assessment, there is no consensus on how this assessment should be performed on observational studies [29]. In the present review, the RoB was assessed by two reviewers using a modified version of Downs and Black [30], adapted to observational studies: test–retest reliability (r = 0.88), inter-rater reliability (r = 0.75), internal consistency (KR-20 = 0.89)), external validity (KR-20: 0.54), and criterion validity (0.89 correlation) [31] (a complete description of the items assessed can be accessed in the Supplementary Information). In this modified version [31] ten items of the original checklist that were not relevant to observational studies were removed (items 8, 13 to 15, 17, 19, and 21 to 24). Accordingly, the adapted checklist consisted of 19 items, including 12 items from the original list (Items 1 to 3, 6, 7, 10, 12, 16, 18, 20, and 25); five items that were modified (Items 4, 5, 9, 26, and 27), and two items created by the authors [30]. Every quality criterion was given a positive (+) sign and one point, if the article provided an adequate description of the item, or a negative sign (−) and a zero point, if the publication did not provide an adequate description or did not address and/or perform the quality criteria for the item. Finally, if an unclear description of the item was provided, an “Unable to determine” was assigned, and zero points were attributed. Consequently, the Downs and Black score ranges were given the corresponding quality levels: excellent (18–19), good (14–17), fair (10–13), or poor (≤9) [30,32].

## 3. Results

### 3.1. Literature Search and Study Identification, Screening and Selection

**Figure 1:**
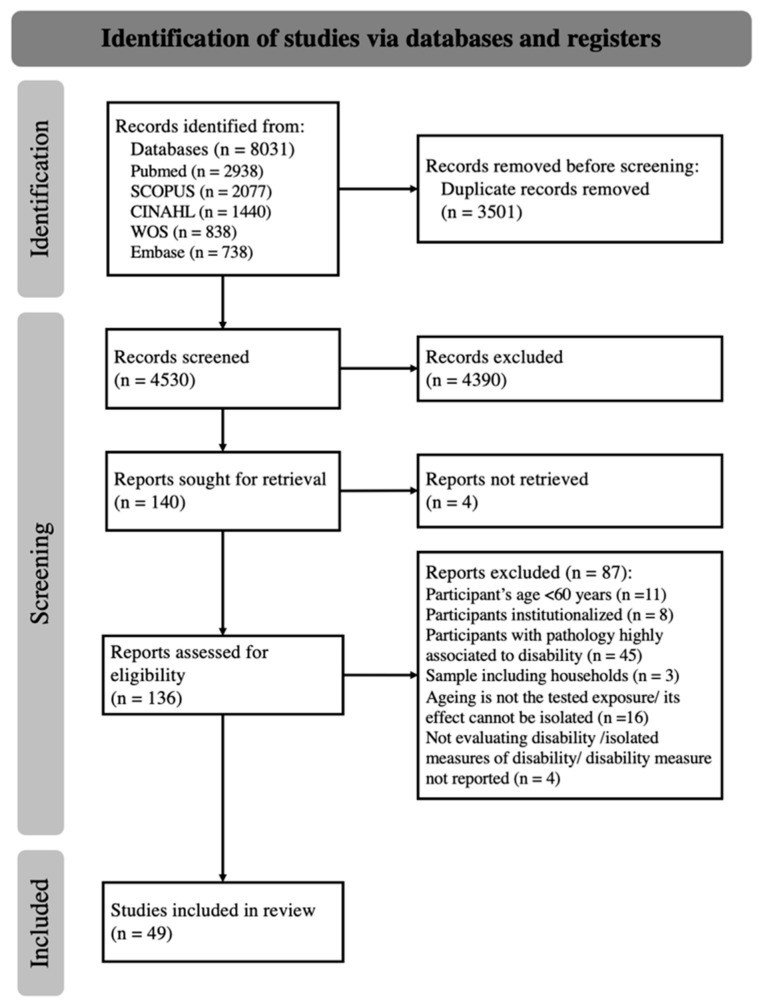
**The** PRISMA 2020 flow diagram for new systematic reviews.

The electronic database search identified a total of 8031 records. From these, 3501 duplicates were removed, resulting in 4530 papers available for the title and abstract screening (Figure 1). The screening of titles and abstracts, performed by two authors with 63% agreement (kappa statistics), identified 136 potentially eligible studies. After discussion, an agreement was reached, or a consensus was established with the third author. After the full-text reading and analysis of the 136 full texts, 49 studies were included in the final analysis [33,34,35,36,37,38,39,40,41,42,43,44,45,46,47,48,49,50,51,52,53,54,55,56,57,58,59,60,61,62,63,64,65,66,67,68,69,70,71,72,73,74,75,76,77,78,79,80,81].

**Figure 2:**
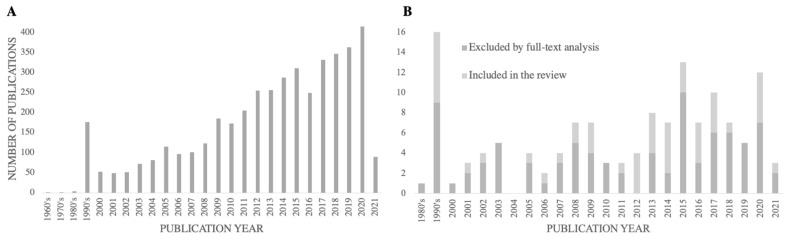
The number of studies retrieved by the database search, excluded by title and abstract screening (**A**), and excluded or included by full-text analysis (**B**), by publication year.

Figure 2 reports the number of studies identified and divided into two groups—excluded by title and abstract, (Figure 2A), and included for full-text analysis, (Figure 2B)—by the year of publication, reflecting the evolution of the search for this thematic throughout the years. Over 36% of the included studies had a publication year equal to or superior to 2015, and the average publication year of the included studies was 2011.

### 3.2. Methodological Quality Assessment

The methodological quality assessment of the 49 included studies presented in Table 1 demonstrates that the Downs and Black total scores ranged from 5 (fair) to 17 (good). Twenty-two of the included studies obtained the classification of good, twenty-four had the classification of fair, and three had the lowest classification class of poor. In the reporting subsection, individual items were completed over 61% of the included sections, excluding the item considering the description of principal covariates, which was only addressed in 39% of the studies. Considering the validity of the studies, the external and the confounding subsection of the internal, were addressed by 37% to 78% of studies, while the bias subsection of internal validity was the focus of most of the studies (94% to 100%). The Power description was the subscale that had the fewest articles fulfilled. Thirty-seven percent reported a formal power calculation for determining the association between age and the outcome measures, and four percent had a sample size reflective of the power calculation.

### 3.3. Content Analysis

The relevant features for study identification and content analysis were summarized according to their classification in Supplementary Tables S1–S3. The included studies have a cross-sectional design, except for five studies that presented a longitudinal design (follow-up time ranging between one and ten years). Sample sizes ranged from 24 [58] to 10 092 [60] older adults, whereas the average of the 49 studies was 1030 participants. Six studies gathered samples which included 100% of older adult women [34,45,55,64,68,73], and one study sampled only men [66]; the percentage of women in the study samples was between 47% [76] and 81.4% [70]. Among the studies that identified the age range, an interval between 60 and 105 years was reported, with a minimal sample-age average of 67.4 years [41] and a maximal of 80 years [79]. To report the indicators associated with age, the included studies opted mainly for bivariate correlations, the Mann–Whitney U test or *t*-test, an Analysis of variance (ANOVA) or the Kruskal–Wallis test, and different types of regression analyses as statistical strategies.

#### 3.3.1. Indicators Expressing a Statistical Association with Age

The studies classified as poor quality identified statistical associations with age for the indicators: lower extremity’s strength/endurance; postural control parameters [36]; fluid and crystallized intelligence; primary, secondary, and tertiary memory [69]; vision and audition ability; and ADL assessed by a scale that has not been reported, see Table S3 [37]. Nevertheless, these three studies presented significant methodological flaws. In particular, none fulfilled the external validity and power sections, and they lacked information in the reporting section (Table 1). Therefore, considering their results for the endorsement of the recommendations could lead to the misinterpretation of study data that hereafter would use the measures assessed and described by them.

Table 2 summarizes the indicators, and respective instruments, that identified significant modifications with age assessed by good and fair quality studies. Indicators assessed by objective measures included the Body Structures, Body Functions, and Environmental Factors. The indicator handgrip strength was used in nine studies [44,45,51,55,58,59,60,66,67], and in eight it presented a statistical association with age, see Tables S1 and S2 [44,45,51,55,59,60,66,67], and from these only six are of good quality, see Table 1 [44,51,59,60,66,67]. This was the indicator that was most frequently identified as having an association with age and has been objectively assessed with an hydraulic or digital dynamometer. The cognitive function indicator, when assessed by the Mini-Mental State Examination, was used in eight studies, see Tables S1 and S2 [39,40,47,64,65,66,70,80], and presented a statistical association with age in seven of those studies [40,47,64,65,66,70,80], of which four are of a good methodology quality, see Table 1 [47,66,70,80]. The gait speed indicator also identified differences with age even when assessed by different instruments [34,47,51,60,67,68,71,73]. The walk test (involving different distances according to the included studies) was the most frequently assessed, being selected by six studies, see Tables S1 and S2 [34,51,60,67,71,73], of which five are of good quality, see Table 1 [51,60,67,71,73]. One of the studies of good quality used the GaitRite™ walkway system [71]. Endurance was also assessed by several tests [33,34,43,45,48,60,67], but the chair-stand test was applied in five studies [34,45,48,60,67], having a statistical association with age in four studies, see Tables S1 and S2 [45,48,60,67], of which three are of a good methodological quality, see Table 1 [48,60,67]. Most of the studies measured the time to complete the task, but Alcock et al., 2015, who did not report an association with aging, used an optoelectronic system [34].

Strength of the lower limbs was assessed by a dynamometer or an isokinetic dynamometer in four studies, see Tables S1 and S2 [34,66,68,73]; reporting the association with age, two of them had a good quality methodology, see Table 1 [66,73]. Cognitive function, memory, and attention were assessed resorting to the Digit from Wechsler Memory Scale-Revised, which was also used and associated with age in four studies, Tables S1 and S2 [40,47,66,80], three having a good quality methodology, see Table 1 [47,66,80].

The remaining indicators, and respective instruments, were assessed and identified as having an association with age in three or fewer included studies.

The indicators that were assessed by subjective measures addressed the Body Functions, Activities and Participation, and Environmental Factors. The most frequently assessed indicator was self-reported health status, evaluated by a single question in eight studies [35,37,39,48,52,53,80,81], although only four reported a significant association with age, see Tables S1 and S2, [39,48,52,53]. The Katz Index of Independence in ADL was used in four studies [39,48,62,74], all of which found a significant relationship between age and ADL (Tables S1 and S2). The Lawton And Brody Instrumental ADL was applied in the same four studies and by Incel et al., 2009 [58] (Table S2), the latter being the only one that has not reported the association of this measure with age. For both instruments reviewed, of the studies that have identified an association with age, three have a good quality methodology, see Table 1 [39,48,62]. Functional ability, considering the ADL, was also assessed by the Barthel Index in three of the included studies, see Tables S1 and S2 [42,63,70], and all of the studies identified a significant association with age, of which two were classified as having a good quality methodology, see Table 1 [63,70]. An identical number of studies applied the Index of Mobility Scale (Rosow and Breslau) to assess functional ability in advanced activities of daily living (AADL), identifying the association with age, see Tables S1 and S2 [38,39,48]; two were of good quality, see Table 1 [39,48].

Considering the objective measures, the remaining self-reported indicators, and respective instruments, were assessed and identified as having an association with age in one or two of the included studies.

#### 3.3.2. Indicators with an Inconclusive Statistical Association with Age

General health status was assessed by the 12-Item Short-Form Health Survey on the Smee et al., 2012 [76] study (Table S2), divided into the physical (physSF-12) and mental (mentSF-12) subscales, with none reporting significant changes with age. The use of the extended scale SF-36 by Incel et al., 2009 [58] also did not demonstrate a significant association with age for the total score (Table S2). Alcock et al., 2015 [34] tested this extended version of the scale by dividing it into Mental and Physical Component summaries, with the latter showing a significant relationship with age (Table S2). The only complete version of this scale presenting a statistical association with age was the SF-20 in the Ghinescu et al., 2014 [52] study, according to a principal component analysis integrating other indicators into the these factors (Table S2). All the studies that assessed general health status by some version of the Short-Form Health Survey were classified with a fair methodological quality, see Tables S1 and S3 [34,52,58,76]. More specifically, the self-assessment of health compared to the past, as assessed by Araújo & Ribeiro, 2011 [37], did not report a statistical association with age (Table S3).

Body composition was subdivided into several indicators as body mass index [42,44,57,60,61], fat or lean mass [66,68] or waist circumference [61], and assessed by different instruments, such as bioelectrical impedance [42,60], a weighing scale [61], a dual-emission X-ray densitometer [66,68], or integrated into the assessment of the Senior fitness test [44,57]. Globally, body composition was assessed by seven studies [42,44,57,60,61,66,68], although two did not report any significant association with age, see Tables S1 and S2 [44,66]. The studies reporting the association with age were classified mainly as a fair methodological quality [42,57,61,68], and fewer were of a good quality [60], Table 1.

The number of medical diagnoses and number of chronic diseases assessed by Araújo & Ribeiro, 2011, see Table S3 [37], and Chen et al., 2012, see Table S2 [42], respectively, did not present a statistical association with age, but Ghinescu et al., 2014, see Table S2 [52], reported an association with age by performing the same statistical procedure applied in the Short-Form Health Survey. Notably, the study of Araújo & Ribeiro, 2011 [37], was classified as having a poor methodological quality and those of Chen et al., 2012 [42] and Ghinescu et al., 2014 [52] as fair (Table 1). Chen et al., 2012 [42], even assessed the frequency of doctor’s visits and hospitalization frequency, but did not present a significant association with age (Table S2). Aligned with this indicator, Hershman et al., 1995 [56], assessed the use of non-prescribed medication and the frequency of medication use by category and found that, with the exception of antihistamines and antidepressants, none had a statistical association with age (Table S2).

Ekström et al., 2016 [49] was the only study assessing the indicators related to environmental factors, but two of the ten indicators, in particular, the perception of physical environment support for performance of daily activities in the home, assessed by the Usability in My Home questionnaire, and the housing satisfaction assessed with a single question, did not present an association with age. These authors also assessed the presence of symptoms assessed by a checklist (excluding head symptoms), which did not present a statistical association with age [49] (Table S2).

Neri et al., 2012 [65], was the only study that explored language performance by assessing the MMSE sentence (verbal fluency–number of words produced and, grammatical complexity–number of phrases or interrelated ideas), which weren’t reported as having a statistical association with age (Table S2).

There were other indicators, and instruments, along with different ranked quality studies, that did not present statistical associations with age. Classified as a good quality study, Uttl et al., 2001 [80], did not find an association with age in self-reported eyesight, hearing, and general health, nor in the assessment of sensation by the Color Vision Screening Inventory (Table S1). Fastame et al., 2020 [50], reported associations with age for perceived psychological well-being, but not for the socially desirable response assessed by the inventory in the Marlowe and Crown Social Desirability Scale (Table S2). Poon et al., 1992 [69], a study with a poor methodological quality, assessed problem-solving ability evaluated by everyday problem resolution, presenting no statistical association with age (Table S3).

There were also three studies in which none of the indicators reported an association with age, particularly, Zunzunegui et al., 2006 [81], classified with a good methodological quality (Table S1); Amarasena et al., 2018 [35], and Incel et al., 2009 [58], both classified with a fair methodological quality (Table S2). The ADL was the indicator assessed by Zunzunegui et al., 2006 [81], using the Index of basic physical activities (Nagi), a set of questions assessing the difficulty for older adults to perform ADL, and self-reported health, as the instruments. Amarasena et al., 2018 [35], assessed self-rated oral and general health through a single question, and both did not present a statistical association with age. Two objective measures were assessed by Incel et al., 2009 [58], namely dynamometry to evaluate the handgrip strength and finger prehension force to assess the hand-tip pinch. Additionally, Incel et al., 2009 [58], used subjective measures (a visual analog scale, the Duruöz hand index, the Lawton and Brody IADL scale and, as stated previously, the Short form 36) to assess self-estimated hand function, functional handicap, manual dexterity, IADL performance, and general health. The authors applied *t*-tests and the Mann–Whitney U test and have not reported any significant association with age.

## 4. Discussion

The present systematic review describes the age-related disability indicators in community-dwelling older adults, which are capable of identifying modifications with age, using the ICF framework as a reference. To our knowledge, this is the first study reporting objective and self-reported indicators for age-related disability components in community-dwelling older adults. To fulfill this purpose, 49 studies assessing the association between aging and the ICF domain indicators, and assessment tools, were included.

Globally, the results of the present study demonstrate an increasing number of studies over the years, which aligns with the increasing socio–economic concerns about the aging population and consequent older adult disability [1,2].

The studies included in the review were exclusively cross-sectional and longitudinal designs, but varied extensively in terms of aims. Study design is of high importance in the gathered instrument’s analysis given that the results of cross-sectional studies should be analyzed with caution due to a “cohort effect” [51], and longitudinal studies have a wide range of follow-up periods. The follow-up times of the included studies differed between one and ten years, with every study reporting a significant association of the implemented measures with age, except Zunzunegui et al., 2006 [81], which had a six-year follow-up. Additionally, Moreira et al. 2016 [62], justified the choice of their two-year follow-up, reporting on previous studies that revealed a two-year correlation between functional decline and anthropometric changes with age. Furuna et al., 1998 [51], assessing handgrip strength, reported that a four-year follow-up study could be too short for detecting longitudinal changes in this indicator, but found modifications according to age group in the gait speed during the same period. Therefore, it seems that the time for the follow-up period is not the preeminent factor for identifying modifications associated with age. Nevertheless, it is important to tailor the period to consider the outcome measure being assessed.

Disability, as described in the ICF [13], is a broad term that encompasses several aspects, namely, impairments in body functions and body structures, limitations in activities, and participation restrictions, denoting the negative aspects of the interaction between an individual and his or her environmental and personal factors. Taking this into consideration, and contemplating the objective or self-reported nature of the measures applied, the indicators and respective instruments were divided into the domains of the ICF to which they were more related. It is important to underline that it was a simple and general classification according to the most preponderant domain assessed by the instrument. Currently, there are defined and updated rules for linking health information to the ICF [82], and although some instruments have been subjected to this process [83,84,85], there is still a need for studies that link instruments, and each of their parts or items, with the domains and categories of the ICF.

The objective measures that have identified significant associations with age in the included studies of this review, integrate the ICF domains of body structures, body functions, and environmental factors. In the body functions domain, there is a wide variability of measures, including measures of general function assessed by, for example, the Senior Fitness Test or Physical Performance Test, and more specific indicators such as handgrip strength or gait speed. These measures, along with cognitive function, were the most frequent disability assessment indicators used and identified as having an association with age in community-dwelling older adults in the included studies. In contrast with the handgrip strength assessment, that was performed in all included studies that identified the association with age by dynamometry [44,45,51,55,59,60,66,67], gait speed was assessed by different instruments such as the walk test [34,51,60,67,71,73], the Timed up and go [34,47,68], and the 8-foot timed walk [48]. Moreover, gait speed was evaluated only using instrumentalization in one study [71]. Appropriately, when assessing an ICF component it is important to define the domain and the different level categories, but also to properly describe the instrument used to allow cross-study comparisons. Ultimately, it would be in the greater interest to standardize a selection of reliable instruments, duly linked to the ICF domains and categories, allowing not only the homogenization of research findings, but also the fulfillment of the health care system’s needs for indicator-based organization. In fact, it would also be decisive to define for each objective instrument the most accurate electronic device to measure the indicators. To assess the objective measures, different electronic devices were selected such as force plates, dynamometers, and optoelectronic systems, among others. These assessments may also be enhanced by wearable devices since they can effectively track older adults remotely [86].

Activities and participation can be assessed by self-report, proxy or caregiver statement, or by direct observation [87], but this review found no objective measures to evaluate this domain. This may be due to the longer time it takes to administer training versus self- or informant reports [87]. Similarly, it can be argued that there is a difficulty in directly observing the execution of the tasks and actions in the older adult’s personal environment. These constraints have resulted in increased efforts to develop self-reported measures in this domain, as evidenced by the number of self-reported instruments found in this review. Yang et al., 2014 [19], who summarized solely self-reported questionnaires on disability designed for, and/or extensively applied to, the older adults, reported that the main assessed domain was the Activities and Participation domain, while using a different methodology than this review. Additionally, these authors [19] described that the most regularly used questionnaires were the Barthel Index, followed by the Lawton and Brody Instrumental Activities of Daily Living Scale, and the Katz Index of Activities of Daily Living. The findings of the present review are consistent with those from Yang et al., 2014 [19], who found that the Katz Index of Activities of Daily Living and the Lawton and Brody Instrumental Activities of Daily Living Scale were identified as having an association with age, in the same four studies [39,48,62,74]. The Barthel Index was also reported by three studies [42,63,70], along with the Index mobility scale (Rosow and Breslau) [38,39,48], all underlining the association of these measures with age. It is essential to note that the studies included have identified the association between age and the different categories of activities as the basic, instrumental, and advanced activities of daily living, capturing the progressive degrees of complexity of the daily conduct of an individual [88]. Although this review reports the assessment of ADL and IADL through subjective measures, novel methods are being developed to assess the dependence on these activities in older adults using machine learning, and wearable or environmental technologies [89,90].

The other self-reported indicators and respective instruments were reported to have an association with age in only one or two study results, with the exception of the self-reported question about overall health status reporting an association with age in four of the eight studies reporting it [39,48,52,53], where it was integrated into the body function’s domain. The literature recommends self-reported health assessment for screening level community-based health studies [91] and patient-centered care in clinical settings [92] and indicates it as a strong predictor of mortality independently of other, more objective health measures [92,93]. However, it should be noted that the studies that included this assessment tool reported different question formulations, with one of them including a thirty-day time frame for the self-assessment of overall health [53].

Considerations regarding the assessment of general health status are also inconclusive, as different versions of the same scale were applied (SF-12, [76], SF-36 [34,58], and SF-20 [52]) and inconsistent results were reported [34,52,58,76]. These results are consistent with the divergent literature appraising this specific instrument. There are reports of its effectiveness in evaluating older adults’ disabilities [94,95], but there are also reports of its limitations on complexity and overestimation of health status results [96].

Finally, this review did not find self-reported measures that assessed the body structure domain, which is consistent with the findings of Yang et al., 2014 [19], because the two reviews did not seek instruments for specific pathologies. 

Both objective and subjective measures identified indicators related to the Body functions and Environmental factors domains. The Body structures domain was only assessed by objective measures and the Activities and Participation domain was only assessed by subjective measures. Independently of the applied nature of the instrument, it is fundamental that all domains of the ICF are assessed to accurately evaluate the presence of disability in an older adult. As indicated by the WHO [13], the disability concept encompasses different domains and all of these need to be assessed to profile an individual’s functioning. Accordingly, each domain needs to be assessed by different indicators considering the multiplicity of constructs included in each domain. Consider the body functions domain, which was defined by thirty-six indicators and assessed by even more instruments. A need for filtering these measures has been identified. Although this review identifies a set of indicators that report modification along with the aging process, it is necessary to define which indicators are more suited to assess the different constructs within each domain.

As the ICF conceptualizes disability as a health experience that occurs in a context, every domain should be assessed to look at medical, individual, social, and environmental influences on functioning and disability [97]. Different categories of older adults’ functioning have been studied [98,99], and some relevant ones have been proposed [100,101]. However, each domain has been assessed independently and, therefore, the integration of the ICF domains into single measures is needed [102]. The usage of technologies in the different instruments to evaluate disability, such as wearables [86] or optoelectronic systems [34], for assessing ICF domains could improve the objectivity of the measures, be cost-effective and decrease assessment time. Even the implementation of the ICF coding itself can be improved by using health technology [103], as its information technology infrastructure for documenting, coding, and reporting has been identified as poor [104]. There were several indicators, and instruments, that presented an association with age only in one of the included studies. Consequently, raising considerations regarding these measures could be premature, and further studies are necessary to assess their association with age.

### Limitations

The results of this review must be interpreted, bearing in mind that gray literature was not included in the search. Nevertheless, to overcome that, and to ensure the retrieval of higher-quality studies, a set of complementary databases were used. Additionally, the summary of the indicators, and respective instruments, comprised the ones identified by the included studies with good and fair methodological qualities. Therefore, when consulting the results of the present review, researchers and health care professionals will be able to easily distinguish the indicators identified by the fair and good-quality studies and interpret their results accordingly.

The indicators and respective instruments summarized in this review identified modifications along with age. However, the report or calculation of the related validity, feasibility, and repeatability of each of the instruments should be addressed in future studies, taking as a reference the, in-development, PRISMA–COSMIN guidelines [105]. This evaluation will allow for the selection of instruments with superior psychometric properties.

In future research, it will be necessary to gather all the instruments that have been formally linked to the ICF to understand which reliable, and frequently applied instruments need to be further linked to allow the comparability of information. According to Cieza et al., 2019 [82], allowing the comparability of data is essential for consistency between decision-makers at all levels of health care organizations.

## 5. Conclusions

This review identified 49 studies, including the outcome assessments used to measure disability in community-dwelling older adults. Most of the included studies reported indicators, and respective assessment tools, that identified significant modifications with age. The conjunct of the studies has identified that self-reported and objective instruments can assess the four domains of the ICF. The indicators assessed by objective measures included Body Structures, Body Functions, and Environmental Factors of the ICF. The most frequent indicators (identified in four or more of the included studies), and respective instruments, that presented a significant association with age, were handgrip strength (dynamometry, *n* = 8), cognitive function (Mini-Mental State examination, *n* = 7), gait speed (walk test, *n* = 6), endurance (Chair stand-test, *n* = 4), strength of the lower limbs (dynamometry *n* = 4) and cognitive function, and memory and attention (Digit from Wechsler Memory Scale-Revised, *n* = 4). The assessment of activities of daily living (ADL) (Katz Index of ADL, *n* = 4 studies) and the instrumental ADL (Lawton and Brody Instrumental ADL, *n* = 4 studies) was carried out using self-reported measures that included activities and participation, but not body structures. However, there were no objective measures assessing the Activity and Participation domain nor the self-reported measures assessing the Body Structures domain. The measures summary gathered by this review may be significant for researchers and health care professionals to select a set indicators, and respective instruments, that effectively identify changes in disability with age, comprehending the four domains of the ICF. Additionally, several indicators can be assessed by electronic devices, or wearable technologies, so establishing the indicators best suited to assess older adults’ health and disability can support the development of new wearables and provide improvements to the existing ones.

## Figures and Tables

**Table 1:** Scores of the methodological quality assessment, by the Modified Downs and Black scale, for the included studies (UD—Unable to determine).

Study Identification	Modified Downs and Black Items and Score																			
	1	2	3	4	5	6	7	8	9	10	11	12	13	14	15	16	17	18	19	Score
Adachi et al., 2015 [33]	+	+	+	+	+	+	+	+	+	UD	−	+	+	+	+	+	+	−	−	15
Alcock et al., 2015 [34]	+	+	−	+	−	+	+	+	+	−	−	+	+	+	+	−	+	+	−	13
Amarasena et al., 2018 [35]	+	+	+	+	−	+	+	+	−	−	+	+	+	+	+	UD	+	−	−	13
Andrade et al., 2018 [36]	+	+	+	−	−	−	−	UD	−	−	−	+	+	+	+	UD	UD	−	−	7
Araújo & Ribeiro, 2011 [37]	+	−	−	−	−	+	−	+	−	−	−	−	+	UD	−	−	+	−	−	5
Arroyo et al., 2007 [38]	+	+	−	+	−	+	+	+	−	+	+	+	+	+	+	UD	+	−	−	13
Barberger et al., 1992 [39]	+	+	+	+	−	+	+	+	−	+	+	+	+	+	+	UD	+	−	−	14
Chang & Dong., 2014 [40]	+	+	+	−	−	+	+	−	−	+	+	+	+	+	+	UD	+	+	−	13
Chao et al., 2013 [41]	+	+	+	+	−	+	−	−	+	+	+	+	+	+	+	UD	−	−	−	12
Chen et al., 2012 [42]	+	+	+	−	−	+	+	+	+	+	+	+	+	+	+	UD	UD	−	−	13
Choudhary, 2020 [43]	+	+	−	−	−	+	+	+	+	−	−	+	+	+	+	UD	+	−	−	11
Chung et al., 2016 [44]	+	+	+	+	+	+	+	+	−	−	−	+	+	+	+	+	−	+	−	14
Confortin & Barbosa, 2015 [45]	+	+	−	+	−	+	−	+	+	−	−	+	+	+	+	UD	+	−	−	11
Desrosiers et al., 2009 [46]	+	+	+	+	−	+	+	+	+	+	+	+	+	+	+	UD	+	+	+	17
Dodge et al., 2008 [47]	+	+	+	+	+	+	+	+	+	+	+	+	+	+	+	+	+	−	−	17
Dong et al., 2014 [48]	+	+	+	+	−	+	+	+	−	−	+	+	+	+	+	UD	+	+	−	14
Ekström et al., 2016 [49]	+	+	−	+	−	+	+	+	+	−	−	+	+	+	+	UD	+	−	−	12
Fastame et al., 2020 [50]	+	+	+	−	+	+	+	UD	+	−	−	+	+	+	+	+	UD	−	−	12
Furuna et al., 1998 [51]	+	+	−	+	+	+	+	+	−	+	+	+	+	+	+	+	−	−	−	14
Ghinescu et al., 2014 [52]	+	+	−	+	−	+	+	+	−	+	+	+	+	+	+	UD	+	−	−	13
Grassi et al., 2020 [53]	+	+	+	+	−	+	+	+	+	+	−	+	+	+	+	UD	+	−	−	14
Hara et al., 2020 [54]	+	+	+	−	−	−	−	−	+	+	−	+	+	+	+	UD	+	+	+	12
Hayashi et al., 2002 [55]	+	+	−	+	−	+	+	+	−	−	−	+	+	+	+	UD	+	+	−	12
Hershman et al., 1995 [56]	+	+	+	+	+	+	+	+	−	UD	UD	+	+	+	−	+	+	−	−	13
Ignasiak et al., 2020 [57]	+	+	+	+	+	+	+	−	+	−	−	+	+	+	+	+	UD	−	−	13
Incel et al., 2009 [58]	+	+	+	+	−	+	+	+	−	−	−	+	+	+	+	UD	UD	−	−	11
Jansen et al., 2008 [59]	+	+	+	+	−	+	+	+	+	−	−	+	+	+	+	UD	+	+	−	14
Makizako et al., 2017 [60]	+	+	+	+	+	+	+	+	+	−	−	+	+	+	+	+	+	+	−	16
Marques et al., 2014 [61]	+	+	−	+	+	+	+	−	+	−	−	+	+	+	+	+	−	+	−	13
Moreira et al., 2016 [62]	+	+	+	+	−	+	+	+	+	+	+	+	+	+	+	UD	+	−	−	15
Nakagawa et al., 2017 [63]	+	+	+	+	+	+	+	+	+	−	−	+	+	+	+	+	UD	+	−	15
Nakamura et al., 2017 [64]	+	+	+	+	−	+	+	+	+	−	−	+	+	+	+	UD	+	−	−	13
Neri et al., 2012 [65]	+	+	+	−	−	+	+	+	+	−	−	+	+	+	+	UD	+	−	−	12
Papadakis et al., 1995 [66]	+	+	+	+	+	+	+	+	+	−	−	+	+	+	+	+	+	+	−	16
Pinheiro et al., 2013 [67]	+	+	+	+	+	+	+	+	+	+	+	+	+	+	+	+	+	−	−	1
Pisciottano et al., 2014 [68]	+	+	+	+	−	+	+	+	−	−	−	+	+	+	+	UD	+	+	−	13
Poon et al., 1992 [69]	+	+	−	−	−	−	−	−	+	−	−	+	+	+	+	UD	−	−	−	7
Prata & Scheicher, 2012 [70]	+	+	+	+	−	+	+	+	+	−	−	+	+	+	+	UD	+	+	−	14
Romero-Ortuno et al., 2009 [71]	+	+	+	+	+	+	+	+	+	−	−	+	+	+	+	+	+	+	−	16
Salvà et al., 2005 [72]	+	+	+	+	−	−	−	+	−	+	+	+	+	+	+	UD	+	−	−	12
Sarvestan et al., 2021 [73]	+	+	+	−	+	+	+	+	+	−	−	+	+	+	+	+	+	+	−	15
Sauvel et al., 1994 [74]	+	+	−	−	−	+	−	+	−	+	+	+	+	+	+	UD	+	−	−	11
Sherman and Reuben, 1998 [75]	+	+	+	+	−	+	+	+	−	−	−	+	+	UD	+	UD	+	−	−	11
Smee et al., 2012 [76]	+	+	+	−	−	+	+	+	−	−	−	−	+	+	+	UD	+	+	−	11
Tomita & Burns, 2013 [77]	+	+	+	+	+	+	−	+	+	+	+	+	+	−	+	+	+	−	−	15
Tomsone et al., 2013 [78]	+	+	+	+	+	+	+	+	+	−	+	+	+	+	+	+	+	−	−	16
Turner et al., 2016 [79]	+	+	+	+	+	+	+	+	+	−	+	+	+	+	+	+	+	−	−	16
Uttl et al., 2001 [80]	+	+	+	−	+	+	+	+	+	+	+	+	+	+	+	+	+	+	−	17
Zunzunegui et al., 2006 [81]	+	+	+	+	+	+	−	+	−	+	+	+	+	+	+	+	+	−	−	15
% positive description by item	100	98	76	73	39	92	80	84	61	37	41	96	100	94	96	39	78	37	4	

**Table 2:** A summary of objective and subjective measures with a significant association with age according to the included study (quality classification ≥ fair); studies of fair quality are indicated with a *, and the others correspond to studies of good quality.

	**ICF**	**Indicator**	**Instrument/Test**	**Included Studies**							
Objective Measures	Body Structures	Body composition	Digital scale/Dual-emission X-ray densitometer	[42] *	[57] *	[60]	[61] *	[68] *			
		Teeth/Masticatory function	Dentist, Ultrasonic diagnostic apparatus	[54] *							
	Body Functions	Physical Performance	Senior Fitness Test	[44]	[57] *	[61] *					
			Physical Performance Test	[66]	[75] *						
			National Institute on Aging Battery	[75] *							
			Continuous-Scale Physical Functional Performance	[76] *							
		Height, reaction time	Jump test	[55] *							
		Mobility	Get up to sit on a chair	[55] *							
			Sit and reach distance	[55] *							
			Pick-up-a-pen test	[67]							
		Endurance	6 min step test	[43] *							
			Shuttle walking test	[33]							
			Chair stand test	[45] *	[48]	[60]	[67]				
		Gait speed	Walk test	[34] *	[51]	[60]	[67]	[71]	[73]		
			Timed up and go	[34] *	[47]	[68] *					
			8-foot timed walk	[48]							
		Gait parameters	Dynamic gait index	[68] *							
			Performance Oriented Mobility Assessment	[72] *	[73]						
		Handgrip strength	Grip test	[44]	[45] *	[51]	[55] *	[59]	[60]	[66]	[67]
		Pinch force	Pinch gauges	[59]							
		Manual speed	Maximum finger-tapping rate test	[51]	[55] *						
		Lower limb Strength	Hip/Knee/Ankle maximum concentric and eccentric voluntary contraction	[34] *	[66]	[68] *	[73]				
		Balance	Four Square Step	[43] *							
			Berg Balance Scale	[63]	[68] *	[70]					
		Static balance	Single leg stance test	[44]	[51]	[55] *					
			Tandem stand	[48]	[67]						
		Postural control parameters	Standing test (eyes open)	[73]							
		Cardiorespiratory function: Respiration rate, Breath-holding, Lung capacity, Diastolic blood pressure	TruZone Peak FlowMeter; sphygmomanometer; number of occurrences in a 60-s period	[42] *							
		Cognitive function	Mini-Mental State Exam	[40] *	[47]	[64] *	[65] *	[66]	[70]	[80]	
		Episodic memory	East Boston Memory Test-Immediate and delayed recall	[40] *							
		Prospective, Retrospective memory	The name, letter, and the check task Buschke Recall 1-3; RAVLT A1-6, B1	[80]							
		Attention and Visuospatial ability	Cancel H, Card sorting	[80]							
			Digit from Wechsler Memory Scale-Revised	[40] *	[47]	[66]	[80]				
		Executive function	11-item Symbol Digit Modalities Test	[40] *							
		Psychomotor and executive function	Trail-making A, B from Halstead-Reitan Neuropsychological Test	[47]	[66]						
		Semantic Fluency	Verbal fluency, Animal and Picture naming	[80]							
		Occupational aptitude	General Aptitude Test Battery	[55] *							
	Environmental Factors	Overall morbidity	Total number of medications	[47]	[56] *						
		Mobility	Use of mobility devices	[49] *							
Subjective measures	Body Functions	Health status	Short Form Health Survey (SF-36), SF20	[34] *	[52] *						
			Elderly Health Assessment Scale	[41] *							
		Relative health	Self-reported question	[39]							
		Overall health status	Self-reported question	[39]	[48]	[52] *	[53]				
		Functioning	World Health Organization Disability Assessment Schedule II	[53]							
		Mental health status	General Health Questionnaire	[55] *							
		Functional independence	Self-reported question	[39]	[49] *						
		Health changes over the last year	Self-reported question	[48]							
		Morbidity	Number of Chronic conditions	[52] *							
		Vision/Audition ability	Self-reported question	[39]	[47] *						
		Dyspnea/Joint pain	Self-reported question	[39]							
		Head symptoms	Checklist	[49] *							
		Mobility	Mobility assessed by a six-level scale	[39]							
			Self-reported question	[77]							
		Psychological functioning	Ryff scales of Psychological Wellbeing	[49] *							
			Psychological Well-Being and Aging Questionnaire	[50] *							
		Sleep	Questionnaire	[79]							
	Activities and Participation	Functional ability/Physical function ADL	Katz Index of ADL	[39]	[48]	[62]	[74] *				
			ADL Staircase	[49] *	[78]						
			Barthel index	[42] *	[63]	[70]					
			Likert-type scale for different tasks	[42] *							
			Self-reported question	[77]							
		Functional ability IADL	Lawton and Brody Instrumental Activities of Daily Living Scale	[39]	[48]	[62]	[74] *				
			Tokyo Metropolitan Institute of Gerontology Index of Competence	[47]	[51]						
			Self-reported question	[77]							
		Functional ability AADL/Mobility	Index of Mobility scale (Rosow and Breslau)	[38] *	[39]	[48]					
			Confinement scale	[74] *							
		Functional ability ADL and IADL	Groningen Activity Restriction Scale	[52] *							
		Basic physical activities	Index of basic physical activities-Nagi	[48]	[38] *						
		Social participation	Assessment of Life Habits	[46]							
		Leisure activities	List of leisure activities	[47]							
	Environmental Factors	Environmental barriers and accessibility problems	Housing Enabler instrument + Functional limitations total number	[49] *							
		Attachment to home	28-item Meaning of home questionnaire	[49] *							
		External control beliefs in relation to home	Housing-related Control Beliefs Questionnaire	[49] *							

## Data Availability

Not applicable.

## Supplementary Materials

The following supporting information can be downloaded at: https://www.mdpi.com/article/10.3390/s22218270/s1, Supplemental Information: Complete search strategy and reference of excluded studies by full-text analysis; Table S1: Characterization and summary of studies included in the review with classification of “Good” in Downs and Black scale, listed by decreasing order of quality score (for those with the same score an alphabetic order was used); Table S2: Characterization and summary of studies included in the review with classification of “Fair” in Downs and Black scale, listed by decreasing order of quality score (for those with the same score an alphabetic order was used); Table S3: Characterization and summary of studies included in the review with classification of “Poor” in Downs and Black scale, listed by decreasing order of quality score (for those with the same score an alphabetic order was used).
