# Supplementary material for: Indicators and Instruments to Assess Components of Disability in Community-Dwelling Older Adults: A Systematic Review
Source: Sensors (Basel). 2022 Oct 28;22(21):8270. doi: 10.3390/s22218270 (PMC9653663; doi:10.3390/s22218270)
Supplement: Supplementary file 1 [file sensors-22-08270-s001.zip › Supplemental Information.pdf]

Supplemental Information

1. Search strategy

This protocol amendment was written a priori, executed before final study selection and data extraction, and followed throughout the review process.

Overview	
Databases	Pubmed<br>Scopus<br>Web of Science<br>CINAHL<br>Embase<br>Subject headings have been customized for each database.<br>Duplicates between databases were removed in EndNote™(Clarivate™), v 20, software.
Search date	April 8th, 2021
Study Design	Observational studies, including cohort and cross-sectional studies
Limits	No date limit (included all studies until April 8th, 2021)<br>Limil study desing to observational studies, including cohort and cross-sectional studies and excluding meta-analyses, reviews, randomized and non-randomized controlled trials, case reports, pilot studies, technical notes, letters, editorials or studies published as conference proceedings<br>No language limits was added to search strategy, but studies with languages other than English, French, Spanish and Portuguese were excluded in title and abstract analysis

Search Strategy	
Population	Community-dwelling older adults
Exposure	Modifications according aging process
Outcome	Disability measures
Study Design	Observational studies, including cohort and cross-sectional studies

Syntax Guide	
MeSH	Medical Subject Heading
exp	Explode a subject heading
*	After a word, a truncation symbol (wildcard) to retrieve plurals or varying endings
#	Truncation symbol for one character
TS	Title and abstract

Pubmed

("aged"[MeSH Terms] OR "aged"[Title/Abstract] OR "elder*"[Title/Abstract] OR "aged, 80 and over"[MeSH Terms] OR "older adult*"[Title/Abstract] OR "older person*"[Title/Abstract] OR "centenarian*"[Title/Abstract] OR "nonagenarian*"[Title/Abstract] OR "octogenarian*"[Title/Abstract])
AND
("independent living"[MeSH Terms] OR "independent living"[Title/Abstract] OR "community dwelling"[Title/Abstract] OR "community-dwellers"[Title/Abstract] OR "community living"[Title/Abstract] OR "aging in place"[Title/Abstract])
AND
("aging"[MeSH Terms] OR "aging"[Title/Abstract] OR "ageing"[Title/Abstract] OR "senescence"[Title/Abstract] OR "Biological Aging"[Title/Abstract])
AND
("Disability Evaluation"[MeSH Terms] OR "Disability Evaluation"[Title/Abstract] OR "disabilit*"[Title/Abstract] OR "disabled persons"[MeSH Terms] OR "disabled person*"[Title/Abstract] OR "handicap*"[Title/Abstract] OR "Functional Status"[MeSH Terms] OR "Functional Status"[Title/Abstract] OR "Functional Independence"[Title/Abstract] OR "functional capacity"[Title/Abstract] OR "functional performance"[Title/Abstract] OR "functional assessment*"[Title/Abstract] OR "physical assessment*"[Title/Abstract] OR "health status"[MeSH Terms] OR “health status”[Title/Abstract] OR "health status indicators"[MeSH Terms] OR "Population Health"[MeSH Terms] OR "Population Health"[Title/Abstract] OR "geriatric assessment"[MeSH Terms] OR "geriatric assessment*"[Title/Abstract])
AND
("observational study"[Publication Type] OR "observational studies as topic"[MeSH Terms] OR "observational stud*"[Title/Abstract] OR "comparative study"[Publication Type] OR "validation study"[Publication Type] OR "Validation Studies as Topic" [MeSH Terms] OR "validation stud*"[Title/Abstract] OR "Journal Article"[Publication Type] OR "cross sectional studies"[MeSH Terms] OR "cross sectional"[Title/Abstract] OR "cross sectional stud*"[Title/Abstract] OR "cohort studies"[MeSH Terms] OR "cohort"[Title/Abstract] OR "cohort stud*"[Title/Abstract] OR "Prospective studies"[MeSH Terms] OR "prospective stud*"[Title/Abstract] OR "Retrospective studies"[MeSH Terms] OR "retrospective stud*"[Title/Abstract] OR "longitudinal studies"[MeSH Terms] OR "Surveys and questionnaires"[MeSH Terms] OR "Feasibility studies"[MeSH Terms] OR "feasibility stud*"[Title/Abstract])
NOT
("randomized controlled trial"[Publication Type] OR "randomized controlled trials as topic"[MeSH Terms] OR "randomized controlled trial"[Title/Abstract] OR "clinical trial"[Publication Type] OR "clinical trial"[Title/Abstract] OR "controlled clinical trial"[Publication Type] OR "controlled clinical trials as topic"[MeSH Terms] OR "controlled clinical trial"[Title/Abstract] OR "Comment"[Publication Type] OR "Letter"[Publication Type] OR "correspondence as topic"[MeSH Terms] OR "Editorial"[Publication Type] OR "Review"[Publication Type] OR "review literature as topic"[MeSH Terms] OR "Systematic review"[Publication Type] OR "Systematic reviews as topic"[MeSH Terms] OR "systematic review"[Title/Abstract] OR "Meta-Analysis"[Publication Type] OR "Meta analysis as Topic"[MeSH Terms] OR "Meta analysis"[Title/Abstract] OR "Meta-analysis as Topic"[MeSH Terms] OR "Guideline"[Publication Type] OR "Practice Guideline"[Publication Type] OR "Practice Guidelines as Topic"[MeSH Terms])

SCOPUS

TITLE-ABS-KEY("aged" OR "elder*" OR "older adult*" OR "older person*" OR "centenarian*" OR "nonagenarian*" OR "octogenarian*")
AND
TITLE-ABS-KEY("independent living" OR "community dwelling" OR "community-dwellers" OR "community living" OR "aging in place")
AND
TITLE-ABS-KEY("aging" OR "ageing" OR "senescence" OR "Biological Aging")
AND
TITLE-ABS-KEY("Disability Evaluation" OR "disabilit*" OR "disabled person*" OR "handicap*" OR "Functional Status" OR "Functional Independence" OR "functional capacity" OR "functional performance" OR "functional assessment*" OR "physical assessment*" OR "health status" OR "Population Health" OR "geriatric assessment*")
AND
(TITLE-ABS-KEY ("observational stud*" OR "cross sectional" OR "cross sectional stud*" OR "cohort" OR "cohort stud*" OR "Prospective stud*" OR "Retrospective stud*" OR "feasibility stud*" OR "validation stud*")
AND NOT
(TITLE-ABS-KEY ("randomized controlled trial" OR "controlled clinical trial" OR "clinical trial" OR "systematic review" OR "meta-analysis"))

Web of Science

TS=("aged" OR "elder*" OR "older adult*" OR "older person*" OR "centenarian*" OR "nonagenarian*" OR "octogenarian*")
AND
TS=("independent living" OR "community dwelling" OR "community-dwellers" OR "community living" OR "aging in place")
AND
TS=("aging" OR "ageing" OR "senescence" OR "Biological Aging")
AND
TS=("Disability Evaluation" OR "disabilit*" OR "disabled person*" OR "handicap*" OR "Functional Status" OR "Functional Independence" OR "functional capacity" OR "functional performance" OR "functional assessment*" OR "physical assessment*" OR "health status" OR "Population Health" OR "geriatric assessment*")
AND TS=("observational stud*" OR "cross-sectional" OR "cross sectional stud*" OR "cohort" OR "cohort stud*" OR "Prospective stud*" OR "Retrospective stud*" OR "feasibility stud*" OR "validation stud*")
NOT TS=("randomized controlled trial" OR "controlled clinical trial" OR "clinical trial" OR "systematic review" OR "meta-analysis")

CINHAL

S1 (MH "aged+") OR (MH "aged, 80 and over+") OR (TI ("aged" OR "elder*" OR "older adult*" OR "older person*" OR "centenarian*" OR "nonagenarian*" OR "octogenarian*") OR AB

("aged" OR "elder*" OR "older adult*" OR "older person*" OR "centenarian*" OR "nonagenarian*" OR "octogenarian*"))
AND
S2 (MH "Community living+") OR (TI ("independent living" OR "community-dwelling" OR "community-dwellers" OR "community living" OR "aging in place") OR AB (“independent living" OR "community-dwelling" OR "community-dwellers" OR "community living" OR "aging in place"))
S3 S1 AND S2
AND
S4 (MH "aging+") OR (TI ("aging" OR "ageing" OR "senescence" OR "Biological aging") OR AB ("aging" OR "ageing" OR "senescence" OR "Biological aging"))
AND
S5 (MH "Disability Evaluation+") OR (MH "Functional Assessment+") OR (MH "Geriatric Assessment+") OR (MH "Functional Status+") OR (MH "Health Status+") OR (MH "Health Status Indicators+") OR (MH "Population health+") OR (TI ("Disability Evaluation" OR "disabilit*" OR "disabled person* OR "handicap*" OR "Functional Status" OR "Functional Independence" OR "functional capacity" OR "functional performance" OR "functional assessment*" OR "physical assessment*" OR "health status" OR "Population Health" OR "geriatric assessment*") OR AB ("Disability Evaluation" OR "disabilit*" OR "disabled person* OR "handicap*" OR "Functional Status" OR "Functional Independence" OR "functional capacity" OR "functional performance" OR "functional assessment*" OR "physical assessment*" OR "health status" OR "Population Health" OR "geriatric assessment*"))
S6 S3 AND S4 AND S5
S7 (PT “Journal Article”) OR (PT “Research”) OR (PT “Research Instrument”) OR (MH “Nonexperimental Studies”) OR (MH” Prospective Studies”) OR (MH “Concurrent Prospective Studies”) OR (MH “Nonparticipant Observation”) OR (MH “Cross Sectional Studies” OR (MH “Observational methods”) OR (MH “Retrospective panel studies”) OR (MH “Validation Studies”) OR (MH “Questionnaires”) OR (MH “Outcomes research”)) OR (TI ("observational stud*" OR "cross sectional" OR "cross sectional stud*" OR "cohort" OR "cohort stud*" OR "Prospective stud*" OR "Retrospective stud*" OR "feasibility stud*" OR "validation stud*") OR AB ("observational stud*" OR "cross sectional" OR "cross sectional stud*" OR "cohort" OR "cohort stud*" OR "Prospective stud*" OR "Retrospective stud*" OR "feasibility stud*" OR "validation stud*"))
NOT
(PT “Randomized Controlled Trials”) OR (PT “Clinical Trial”) OR (PT “Commentary”) OR (PT “Editorial”) OR (PT “Letter”) OR (PT “Meta Analysis”) OR (PT “Practice Guidelines”) OR (PT “Review”) OR (PT “Systematic Review”) OR (MH “Randomized Controlled Trials”) OR (MH “Clinical Trials”) OR (MH “Meta Analysis”) (MH “Scoping Review”) OR (MH “Systematic Review”) OR (MH “Literature Review”) OR MH (“Practice Guidelines”) OR (TI ("randomized controlled trial" OR "controlled clinical trial" OR "clinical trial" OR "systematic review" OR "meta-analysis") OR AB ("randomized controlled trial" OR "controlled clinical trial" OR "clinical trial" OR "systematic review" OR "meta-analysis"))

EMBASE

('aged'/exp OR 'very elderly'/exp)
OR
('aged':ab,ti OR 'elder*':ab,ti OR 'older adult*':ab,ti OR 'older person*':ab,ti OR 'centenarian*':ab,ti OR 'nonagenarian*':ab,ti OR 'octogenarian*':ab,ti)

AND
('independent living/exp' OR 'community living'/exp OR 'community dwelling person'/exp)
OR
('independent living':ab,ti OR 'community-dwelling':ab,ti OR 'community-dwellers':ab,ti OR 'community living':ab,ti OR 'aging in place':ab,ti)
AND
('aging'/exp)
OR
('aging':ab,ti OR 'ageing':ab,ti OR 'senescence':ab,ti OR 'Biological aging':ab,ti)
AND
('disability/exp' OR 'disabled person'/exp OR 'functional status'/exp OR 'health status/exp' OR 'health status indicator'/exp OR 'population health'/exp OR 'functional assessment/exp' OR 'geriatric assessment'/exp OR 'physical disability'/exp)
OR
('disabilit*':ab,ti OR 'disability evaluation':ab,ti OR 'disabled person*':ab,ti OR 'handicap*':ab,ti OR 'functional status':ab,ti OR 'functional independence':ab,ti OR 'functional capacity':ab,ti OR 'functional performance':ab,ti OR 'functional assessment*':ab,ti OR 'physical assessment*':ab,ti OR 'health status*':ab,ti OR 'population health':ab,ti OR 'geriatric assessment':ab,ti)
AND
('cohort analysis'/de OR 'comparative study'/de OR 'cross sectional study'/de OR 'diagnostic test accuracy study'/de OR 'feasibility study'/de OR 'human'/de OR 'longitudinal study'/de OR 'observational study'/de OR 'outcomes research'/de OR 'prospective study'/de OR 'questionnaire'/de OR 'retrospective study'/de OR 'validation study'/de) OR ('Prospective Study'/exp OR 'Cross sectional study'/exp OR 'Observational study'/exp OR 'Cohort analysis'/exp OR 'Retrospective study'/exp OR 'Validation Study'/exp OR 'Longitudinal study'/exp OR 'Questionnaire'/exp OR 'Outcomes research'/exp OR 'Feasibility study'/exp OR 'Diagnostic test accuracy'/exp)
OR
('observational stud*':ab,ti OR 'cross sectional':ab,ti OR 'cross sectional stud*':ab,ti OR 'cohort':ab,ti OR 'cohort stud*':ab,ti OR 'cohort analysis':ab,ti OR 'prospective stud*':ab,ti OR 'retrospective stud*':ab,ti OR 'feasibility stud*':ab,ti OR 'validation stud*':ab,ti)
NOT
'clinical article'/de OR 'clinical trial'/de OR 'controlled clinical trial'/de OR 'controlled study'/de OR 'human experiment'/de OR 'human tissue'/de OR 'interview'/de OR 'major clinical study'/de OR 'meta analysis'/de OR 'model'/de OR 'nonhuman'/de OR 'practice guideline'/de OR 'randomized controlled trial'/de OR 'randomized controlled trial topic'/de OR 'semi structured interview'/de OR 'systematic review'/de OR 'telephone interview'/de OR 'randomized controlled trial'/exp OR 'controlled study'/exp OR 'clinical trial'/exp OR 'letter'/exp OR 'editorial'/exp OR 'review'/exp OR 'meta analysis'/exp OR 'practice guideline'/exp OR 'randomized controlled trial':ab,ti OR 'controlled clinical trial':ab,ti OR 'clinical trial':ab,ti OR 'systematic review':ab,ti OR 'meta analysis':ab,ti

2. Assessment of methodologic quality

Modified version of Downs and Black adapted to observational studies (Rollo, S.; Antsygina, O.; Tremblaya, M.S. The whole day matters: Understanding 24-hour movement guideline adherence and relationships with health indicators across the lifespan. J Sport Health Sci. 2020, 9, 493–510, doi:10.1016/j.jshs.2020.07.004.)

**Reporting**
1. Is the hypothesis/aim/objective of the study clearly described?
2. Are the main outcomes to be measured clearly described in the Introduction or Methods section?
3. Are the characteristics of the participants included in the study clearly described?
4. Is the study design clearly described (i.e., cross-sectional vs. prospective; if prospective, time of assessments)?
5. When appropriate, were principal covariates clearly described?
6. Are the main findings of the study clearly described?
7. Does the study provide estimates of the random variability in the data for the main outcomes?
8. Have the characteristics of participants lost to follow-up and/or with missing data been described?
9. Have actual probability values been reported (e.g., 0.035 rather than <0.05) for the main outcomes except where the probability value is <0.001?
**External validity**
10. Were the subjects asked to participate in the study representative of the entire population from which they were recruited?
11. Were those subjects who were prepared to participate representative of the entire population from which they were recruited?
**Internal validity bias**
12. If any of the results of the study were based on "data dredging", was this made clear?
13. Were the exposure(s) of interest measured prior to the outcome(s) being measured?
14. Were the statistical tests used to assess the main outcomes appropriate?
15. Were the main exposure and outcome measures used accurate (valid and reliable)?
**Internal validity confounding (selection bias)**
16. When appropriate, was there adequate adjustment for confounding (i.e., covariates) in the analyses from which the main findings were drawn?
17. Were losses of participants to follow-up and/or with missing data taken into account?
**Power**
18. Did the study report a formal power calculation for determining the association between exposure(s) and outcome(s) variables?
19. Was the sample size used for analyses reflective of the power calculation?

3. Excluded Studies after full text analysis

The majority of the studies were excluded since included in the sample participants with pathology highly associated to disability (1-45), 11 because the sample included participants under the age of 60 years (46-56), 8 a sample with institutionalized patients (57-64), and 3 including households (65-67). 16 studies were excluded because the ageing process was not the tested exposure, or its effect could not be isolated, or the statistical descriptive values were not provided (68-83). Lacking evaluating of disability, or isolated measures of disability, or disability measure not reported was the criteria to exclude 4 studies (84-87).

1. Anstey KJ, Smith GA. Interrelationships among biological markers of aging, health, activity, acculturation, and cognitive performance in late adulthood. Psychology and aging. 1999;14(4).
2. Atkinson HH, Cesari M, Kritchevsky SB, Penninx BWJH, Fried LP, Guralnik JM, et al. Predictors of combined cognitive and physical decline. Journal of the American Geriatrics Society. 2005;53(7).
3. Buchman AS, Wilson RS, Yu L, James BD, Boyle PA, Bennett DA. Total daily activity declines more rapidly with increasing age in older adults. Archives of gerontology and geriatrics. 2014;58(1).
4. Burns RA, Browning C, Kendig HL. Living well with chronic disease for those older adults living in the community. International psychogeriatrics. 2017;29(5).
5. Busch TdA, Duarte YA, Nunes DP, Lebrão ML, Naslavsky MS, Rodrigues AdS, et al. Factors associated with lower gait speed among the elderly living in a developing country: a cross-sectional population-based study. BMC geriatrics. 2015;15.
6. Christensen H, Jorm AF, Henderson AS, Mackinnon AJ, Korten AE, Scott LR. The relationship between health and cognitive functioning in a sample of elderly people in the community. Age and ageing. 1994;23(3).
7. Connolly D, Garvey J, McKee G. Factors associated with ADL/IADL disability in community dwelling older adults in the Irish longitudinal study on ageing (TILDA). Disability and rehabilitation. 2017;39(8).
8. Dale W, Kotwal AA, Shega JW, Schumm LP, Kern DW, Pinto JM, et al. Cognitive Function and its Risk Factors Among Older US Adults Living at Home. Alzheimer disease and associated disorders. 2018;32(3).
9. Doyev R, Axelrod R, Keinan-Boker L, Shimony T, Goldsmith R, Nitsan L, et al. Energy Intake Is Highly Associated with Handgrip Strength in Community-Dwelling Elderly Adults. The Journal of nutrition. 2021;151(5).
10. Fabre JM, Wood RH, Cherry KE, Su LJ, Cress ME, King CM, et al. Age-related deterioration in flexibility is associated with health-related quality of life in nonagenarians. Journal of geriatric physical therapy (2001). 2007;30(1).
11. Ford AB, Haug MR, Stange KC, Gaines AD, Noelker LS, Jones PK. Sustained personal autonomy: a measure of successful aging. Journal of aging and health. 2000;12(4).
12. Forrest KYZ, Zmuda JM, Cauley JA. Patterns and correlates of muscle strength loss in older women. Gerontology. 2007;53(3).
13. Fujiwara Y, Yoshida H, Amano H, Fukaya T, Liang J, Uchida H, et al. Predictors of improvement or decline in instrumental activities of daily living among community-dwelling older Japanese. Gerontology. 2008;54(6).
14. García-Esquinas E, Ortolá R, Prina M, Stefler D, Rodríguez-Artalejo F, Pastor-Barriuso R. Trajectories of Accumulation of Health Deficits in Older Adults: Are There Variations According to Health Domains? Journal of the American Medical Directors Association. 2019;20(6).
15. Gell NM, Wallace RB, LaCroix AZ, Mroz TM, Patel KV. Mobility device use in older adults and incidence of falls and worry about falling: findings from the 2011-2012 national health and aging trends study. Journal of the American Geriatrics Society. 2015;63(5).
16. Gill TM, Guo Z, Allore HG. Subtypes of disability in older persons over the course of nearly 8 years. Journal of the American Geriatrics Society. 2008;56(3).
17. Han L, Allore H, Murphy T, Gill T, Peduzzi P, Lin H. Dynamics of functional aging based on latent-class trajectories of activities of daily living. Annals of epidemiology. 2013;23(2).

18. Jang Y, Haley WE, Mortimer JA, Small BJ. Moderating effects of psychosocial attributes on the association between risk factors and disability in later life. Aging & mental health. 2003;7(3).
19. Jung H-W, Jang I-Y, Lee CK, Yu SS, Hwang JK, Jeon C, et al. Usual gait speed is associated with frailty status, institutionalization, and mortality in community-dwelling rural older adults: a longitudinal analysis of the Aging Study of Pyeongchang Rural Area. Clinical interventions in aging. 2018;13.
20. Kabayama M, Mikami H, Kamide K. Multidimensional factors associated with the loss of independence: A population-based longitudinal study. Geriatrics & gerontology international. 2018;18(3).
21. Kim S, Han D, Lee J. PREVALENCE AND CORRELATES OF IMPAIRMENTS IN ACTIVITIES OF DAILY LIVING IN OLDER KOREANS: COMPARISON OF YOUNG-OLD AND OLD-OLD. Journal of Men's Health. 2019;15(3):1-10.
22. Kim S, Kim M, Han D. Incidence Rates of Disability and Its Associated Factors among Korean Community-Dwelling Older Adults. Iranian journal of public health. 2020;49(9).
23. Mueller-Schotte S, Zuithoff NPA, Schouw YTvd, Schuurmans MJ, Bleijenberg N. Trajectories of Limitations in Instrumental Activities of Daily Living in Frail Older Adults With Vision, Hearing, or Dual Sensory Loss. The journals of gerontology Series A, Biological sciences and medical sciences. 2019;74(6).
24. Murayama H, Liang J, Shaw BA, Botoseneanu A, Kobayashi E, Fukaya T, et al. Socioeconomic Differences in Trajectories of Functional Capacity Among Older Japanese: A 25-Year Longitudinal Study. Journal of the American Medical Directors Association. 2020;21(6).
25. Nüesch E, Pablo P, Dale CE, Prieto-Merino D, Kumari M, Bowling A, et al. Incident disability in older adults: prediction models based on two British prospective cohort studies. Age and ageing. 2015;44(2).
26. Piguet O, Grayson DA, Broe GA, Tate RL, Bennett HP, Lye TC, et al. Normal aging and executive functions in "old-old" community dwellers: poor performance is not an inevitable outcome. International psychogeriatrics. 2002;14(2).
27. Piguet O, Ridley L, Grayson DA, Bennett HP, Creasey H, Lye TC, et al. Are MRI white matter lesions clinically significant in the 'old-old'? Evidence from the Sydney Older Persons Study. Dementia and geriatric cognitive disorders. 2003;15(3).
28. Pinto JM, Neri AL. Factors associated with low life life satisfaction in community-dwelling elderly: FIBRA Study. Cadernos de saude publica. 2013;29(12).
29. Rodgers V, Neville S, Grow SL. Health, functional ability and life satisfaction among older people 65 years and over: a cross-sectional study. Contemporary nurse. 2017;53(3).
30. Schmitt FA, Phillips BA, Cook YR, Berry DT, Wekstein DR. Self report on sleep symptoms in older adults: correlates of daytime sleepiness and health. Sleep. 1996;19(1).
31. Siordia C. Intersecting self-reportedmobility and gait speedto create a multi-dimensional measure of ambulation: The "Ambulation Speed-ENendurance" (ASE) Typology. The Journal of frailty & aging. 2015;4(2).
32. Smagula SF, Harrison S, Cauley JA, Ancoli-Israel S, Cawthon PM, Cummings S, et al. Determinants of Change in Objectively Assessed Sleep Duration Among Older Men. American journal of epidemiology. 2017;185(10).
33. Steffen TM, Hacker TA, Mollinger L. Age- and gender-related test performance in community-dwelling elderly people: Six-Minute Walk Test, Berg Balance Scale, Timed Up & Go Test, and gait speeds. Physical therapy. 2002;82(2).
34. Stineman MG, Zhang G, Kurichi JE, Zhang Z, Streim JE, Pan Q, et al. Prognosis for functional deterioration and functional improvement in late life among community-dwelling persons. PM & R : the journal of injury, function, and rehabilitation. 2013;5(5).
35. Strotmeyer ES, Winger ME, Cauley JA, Boudreau RM, Cusick D, Collins RF, et al. Normative Values of Muscle Power using Force Plate Jump Tests in Men Aged 77-101 Years: The Osteoporotic Fractures in Men (MrOS) Study. The journal of nutrition, health & aging. 2018;22(10).
36. Tassino M, Campos TF, Guerra RO. Homocysteine (Hcy) and cognitive performance in a population sample of elderly Brazilians. Archives of gerontology and geriatrics. 2009;48(2).
37. Thaler-Kall K, Peters A, Thorand B, Grill E, Autenrieth CS, Horsch A, et al. Description of spatio-temporal gait parameters in elderly people and their association with history of falls: results of the population-based cross-sectional KORA-Age study. BMC geriatrics. 2015;15.
38. Tiedemann A, Sherrington C, Lord SR. Physiological and psychological predictors of walking speed in older community-dwelling people. Gerontology. 2005;51(6).
39. Trevisan C, Rizzuto D, Sergi G, Maggi S, Welmer A-K, Vetrano DL. Peak expiratory flow, walking speed and survival in older adults: An 18-year longitudinal population-based study. Experimental gerontology. 2020;135.

40. Ward RE, Caserotti P, Faulkner K, Boudreau RM, Zivkovic S, Lee C, et al. Peripheral nerve function and lower extremity muscle power in older men. Archives of physical medicine and rehabilitation. 2014;95(4).
41. Wu SC, Leu SY, Li CY. Incidence of and predictors for chronic disability in activities of daily living among older people in Taiwan. Journal of the American Geriatrics Society. 1999;47(9).
42. Xue Q-L, Beamer BA, Chaves PHM, Guralnik JM, Fried LP. Heterogeneity in rate of decline in grip, hip, and knee strength and the risk of all-cause mortality: the Women's Health and Aging Study II. Journal of the American Geriatrics Society. 2010;58(11).
43. Xue Q-L, Guralnik JM, Beamer BA, Fried LP, Chaves PHM. Monitoring 6-month trajectory of grip strength improves the prediction of long-term change in grip strength in disabled older women. The journals of gerontology Series A, Biological sciences and medical sciences. 2015;70(3).
44. Yeh S-CJ, Liu Y-Y. Influence of social support on cognitive function in the elderly. BMC health services research. 2003;3(1).
45. Yip AM, Kephart G, Rockwood K. Linkage of the Canadian Study of Health and Aging to provincial administrative health care databases in Nova Scotia. International psychogeriatrics. 2001;13 Supp 1.
46. Brady CB, 3rd AS, Gaziano M. Effects of age and hypertension status on cognition: the Veterans Affairs Normative Aging Study. Neuropsychology. 2005;19(6).
47. Castellanos-Perilla N, Borda MG, Fernández-Quilez Á, Aarsland V, Soennesyn H, Cano-Gutiérrez CA. Factors associated with functional loss among community-dwelling Mexican older adults. Biomedica:revista del Instituto Nacional de Salud. 2020;40(3).
48. Eslinger PJ, Swan GE, Carmelli D. Changes in Mini-Mental State Exam in community-dwelling older persons over 6 years: relationship to health and neuropsychological measures. Neuroepidemiology. 2003;22(1).
49. Goble DJ, Baweja HS. Postural sway normative data across the adult lifespan: Results from 6280 individuals on the Balance Tracking System balance test. Geriatrics & gerontology international. 2018;18(8).
50. Lee W-J, Liang C-K, Peng L-N, Chiou S-T, Chen L-K. Protective factors against cognitive decline among community-dwelling middle-aged and older people in Taiwan: A 6-year national population-based study. Geriatrics & gerontology international. 2017;17 Suppl 1.
51. Li C, Layman AJ, Carey JP, Agrawal Y. Epidemiology of vestibular evoked myogenic potentials: Data from the Baltimore Longitudinal Study of Aging. Clinical neurophysiology : official journal of the International Federation of Clinical Neurophysiology. 2015;126(11).
52. Manckoundia P, Thomas F, Buatois S, Guize L, Jégo B, Aquino J-P, et al. Impact of clinical, psychological, and social factors on decreased Tinetti test score in community-living elderly subjects: a prospective study with two-year follow-up. Medical science monitor : international medical journal of experimental and clinical research. 2008;14(6).
53. Paúl C, Ribeiro O, Santos P. Cognitive impairment in old people living in the community. Archives of gerontology and geriatrics. 2010;51(2).
54. Saito A, Wakasa M, Kimoto M, Ishikawa T, Tsugaruya M, Kume Y, et al. Age-related changes in muscle elasticity and thickness of the lower extremities are associated with physical functions among community-dwelling older women. Geriatrics & gerontology international. 2019;19(1).
55. Smith AR, Chen C, Clarke P, Gallagher NA. Trajectories of Outdoor Mobility in Vulnerable Community-Dwelling Elderly: The Role of Individual and Environmental Factors. Journal of aging and health. 2016;28(5).
56. Tang Z, Jiang J, Futatsuka M. An evaluation of transition in functional states among the elderly in Beijing, China. Environmental health and preventive medicine. 2002;7(5).
57. Bravell ME, Stig Berg BM. Health, functional capacity, formal care, and survival in the oldest old: a longitudinal study. Archives of gerontology and geriatrics. 2008;46(1).
58. Cohen-Mansfield J, Shmotkin D, Blumstein Z, Shorek A, Eyal N, Hazan H, et al. The old, old-old, and the oldest old: continuation or distinct categories? An examination of the relationship between age and changes in health, function, and wellbeing. International journal of aging & human development. 2013;77(1).
59. Fastame MC, Cavallini E. Working Memory Functions in Healthy Elderly People: The Impact of Institutionalization and Advancing Age on Mnestic Efficiency. Clinical Gerontologist. 2011;34(3):207–19.
60. Ferraro OE, Guaita A, Villani S. Cognitive, physical and disability trajectories in community-dwelling elderly people. Aging clinical and experimental research. 2021;33(10).

61. Freeman S, Kurosawa H, Ebihara S, Kohzuki M. Understanding the oldest old in northern Japan: an overview of the functional ability and characteristics of centenarians. Geriatrics & gerontology international. 2010;10(1).
62. Mustafaoğlu R, Unver B, Karatosun V. Evaluation of stair climbing in elderly people. Journal of back and musculoskeletal rehabilitation. 2015;28(3).
63. Shubert TE, Schrodt LA, Mercer VS, Busby-Whitehead J, Giuliani CA. Are scores on balance screening tests associated with mobility in older adults? Journal of geriatric physical therapy (2001). 2006;29(1).
64. Vellas BJ, Rubenstein LZ, Ousset PJ, Faisant C, Kostek V, Nourhashemi F, et al. One-leg standing balance and functional status in a population of 512 community-living elderly persons. Aging (Milan, Italy). 1997;9(1-2).
65. Prince MJ, Guerra M, Huang Y, Lloyd-Sherlock P, Sosa AL, Uwakwe R, et al. Health and economic correlates of autonomy among older people in Peru, Mexico and China: The 10/66 INDEP study. Wellcome Open Research. 2018;3(57).
66. The VN, Assanangkornchai S. Functional Disability among Community-Dwelling Older People: Prevalence and Associated Factors in Thanh Hoa City and Hoang Hoa District, Vietnam. International Journal of Gerontology. 2020;14(3):222-6.
67. Willie-Tyndale D, Holder-Nevins D, Mitchell-Fearon K, James K, Laws H, Waldron NK, et al. Participation in Social Activities and the Association with Socio-Demographic and Health-Related Factors among Community-Dwelling Older Adults in Jamaica. Journal of cross-cultural gerontology. 2016;31(4).
68. Baloh RW, Corona S, Jacobson KM, Enrietto JA, Bell T. A prospective study of posturography in normal older people. Journal of the American Geriatrics Society. 1998;46(4).
69. Inzitari M, Pozzi C, Ferrucci L, Chiarantini D, Rinaldi LA, Baccini M, et al. Subtle neurological abnormalities as risk factors for cognitive and functional decline, cerebrovascular events, and mortality in older community-dwelling adults. Archives of internal medicine. 2008;168(12).
70. McAuliffe MJ, Schluter PJ, Jamieson HA. An epidemiological profile of communication disability among older adults with complex needs: A national cross-sectional study. International journal of speech-language pathology. 2019;21(6).
71. Nikula S, Jylhä M, Bardage C, Deeg DJH, Gindin J, Minicuci N, et al. Are IADLs comparable across countries? Sociodemographic associates of harmonized IADL measures. Aging clinical and experimental research. 2003;15(6).
72. Purath J, Buchholz SW, Kark DL. Physical fitness assessment of older adults in the primary care setting. Journal of the American Academy of Nurse Practitioners. 2009;21(2).
73. Riebe D, Blissmer BJ, Greaney ML, Garber CE, Lees FD, Clark PG. The relationship between obesity, physical activity, and physical function in older adults. Journal of aging and health. 2009;21(8).
74. Rudberg MA, Parzen MI, Leonard LA, Cassel CK. Functional limitation pathways and transitions in community-dwelling older persons. The Gerontologist. 1996;36(4).
75. Sallinen J, Mänty M, Leinonen R, Kallinen M, Törmäkangas T, Heikkinen E, et al. Factors associated with maximal walking speed among older community-living adults. Aging clinical and experimental research. 2011;23(4).
76. Simonsick EM, Kasper JD, Phillips CL. Physical disability and social interaction: factors associated with low social contact and home confinement in disabled older women (The Women's Health and Aging Study). The journals of gerontology Series B, Psychological sciences and social sciences. 1998;53(4).
77. Stessman J, Rottenberg Y, Fischer M, Hammerman-Rozenberg A, Jacobs JM. Handgrip Strength in Old and Very Old Adults: Mood, Cognition, Function, and Mortality. Journal of the American Geriatrics Society. 2017;65(3).
78. Woo J, Ho SC, Sham A. Longitudinal changes in body mass index and body composition over 3 years and relationship to health outcomes in Hong Kong Chinese age 70 and older. Journal of the American Geriatrics Society. 2001;49(6).
79. Yu R, Wong M, Chang B, Lai X, Lum CM, Auyeung TW, et al. Trends in activities of daily living disability in a large sample of community-dwelling Chinese older adults in Hong Kong: an age-period-cohort analysis. BMJ open. 2016;6(12).
80. Chen C-M, Su Y-Y, Mullan J, Huang M-S, Chiu H-C. Trajectories of disability and their relationship with health status and social service use. Experimental aging research. 2015;41(3).
81. Madeira T, Peixoto-Plácido C, Sousa-Santos N, Santos O, Alarcão V, Nicola PJ, et al. Geriatric Assessment of the Portuguese Population Aged 65 and Over Living in the Community: The PEN-3S Study. Acta medica portuguesa. 2020;33(7-8).

82. Whelan EJ. Being old in Ireland: a fit state? Irish medical journal. 2009;102(7).
83. Williams ID, O'Doherty LJ, Mitchell GK, Williams KE. Identifying unmet needs in older patients--nurse-GP collaboration in general practice. Australian family physician. 2007;36(9).
84. Branch LG, Horowitz A, Carr C. The implications for everyday life of incident self-reported visual decline among people over age 65 living in the community. The Gerontologist. 1989;29(3).
85. Hébert R, Brayne C, Spiegelhalter D. Incidence of functional decline and improvement in a community-dwelling, very elderly population. American journal of epidemiology. 1997;145(10).
86. Luthy C, Cedraschi C, Allaz A-F, Herrmann FR, Ludwig C. Health status and quality of life: results from a national survey in a community-dwelling sample of elderly people. Quality of life research : an international journal of quality of life aspects of treatment, care and rehabilitation. 2015;24(7).
87. Montero-Odasso M, Speechley M, Muir-Hunter SW, Pieruccini-Faria F, Sarquis-Adamson Y, Hachinski V, et al. Dual decline in gait speed and cognition is associated with future dementia: evidence for a phenotype. Age and Ageing. 2020;49(6):995-1002.
