# Supplementary material for: Indicators and Instruments to Assess Components of Disability in Community-Dwelling Older Adults: A Systematic Review
Source: Sensors (Basel). 2022 Oct 28;22(21):8270. doi: 10.3390/s22218270 (PMC9653663; doi:10.3390/s22218270)
Supplement: Supplementary file 1 [file sensors-22-08270-s001.zip › Table_S1.pdf]

Table S1. Characterization and summary of studies included in the review with classification of “Good” in Downs and Black scale, listed by decreasing order of quality score (for those with same score alphabetic order was used).

Notes: AG – age group, ANOVA - Analysis of variance; M±SD – Mean plus Standard Deviation; NR – non reported; NS – non significant; * - study design non reported in the respective study, and thus classified by the reviewers; † - indicators with significative change or association with age.

ID	**Study Design** (Follow-up time) **Sample Size**	**Age** M±SD [range] % female	**Indicators** Objective & Self-reported	**Instrument**	**Main statistical strategy used to assess the influence of age**	**Significative change/ association with age**
**Desrosiers et al., 2009 [46]** (Canada)	Cross-sectional<br>n=350<br>AG<br>(65–69)<br>n=70<br>(70–74)<br>n=70<br>(75–79)<br>n=70<br>(80–84)<br>n=70<br>(85+)<br>n=70	AG<br>(65–69)<br>67.2 ± 1.5<br>(70–74)<br>71.9 ± 1.3<br>(75–79)<br>76.9 ± 1.4<br>(80–84)<br>81.9 ± 1.5<br>(85+)<br>87.9 ± 2.4<br>50%	*Self-reported measure:*<br>1. Social participation assessed by questionnaire	1. Assessment of Life Habits (LIFE-H)	ANOVA of the LIFE-H scores were performed with the five age groups.<br>Bivariate correlations were used to identify statistically significant variables associated with participation. Independent variables, including demographic, health-related and environmental characteristics that were correlated at p<0.15 with the participation scores were put in each of the two main models (LIFE-H daily activities subscore and LIFE-H social roles subscore) as well as each domain of the LIFE-H. Ordinal variables with more than two levels were treated as continuous variables.	Daily activities: Nutrition p=0.97; Fitness p=0.06; Personal care **p<0.001**†; Communication **p<0.001**†; Housing **p=0.003**†; Mobility **p<0.001**†; Daily activities subscore **p<0.001**† Social roles: Responsibilities p=0.09; Interpersonal relationships p=0.46; Community life **p<0.001**†; Leisure **p<0.001**†; Social roles subscore **p<0.001**†<br>Total score **p<0.001**†<br>Daily activities Age β=0.02, $CumR^2$=0.12 **p<0.001**†<br>Personal care Age β=0.01, $CumR^2$=0.11, **p<0.001**†<br>Communication Age β=0.19, $CumR^2$=0.06, **p<0.001**†<br>Housing Age β=0.03, $CumR^2$=0.06, **p<0.001**†<br>Mobility Age β=0.06, $CumR^2$=0.14, **p<0.001**†<br>Social roles Age β=0.02, $CumR^2$=0.06, **p<0.001**†<br>Community life Age β=0.04, $CumR^2$=0.07, **p<0.001**†
**Dodge et al., 2008 [47]** (Japan)	Cross-sectional<br>n=303<br>AG<br>(65–69)<br>n=68<br>(70–74)<br>n=62<br>(75–79)<br>n=77<br>(80–84)<br>n=53<br>(85+)<br>n=43	76.1 ± 6.9<br>[65, 96]<br>AG<br>(65–69)<br>51.5%<br>(70–74)<br>41.9%<br>(75–79)<br>55.8%<br>(80–84)<br>49.1%<br>(85+)<br>45.2%	*Self-reported measures:*<br>1. Vision or hearing impairment assessed by questionnaire<br>2. Instrumental Activities of Daily Living (IADL) disabilities assessed by questionnaire<br>3. Leisure activities assessed by questionnaire<br>*Objective measures:*<br>4. Mobility assessed by time to complete Timed up and go test (TUG)<br>5. Morbidity measured by medication<br>6. Cognitive function assessed by tests	1. ‘‘what is your visual ability with your visual aid?’’; ‘‘what is your hearing ability with your hearing aid?’’<br>2. Tokyo Metropolitan Institute of Gerontology Index of Competence; questions about telephone use, medication management, and cleaning rooms<br>3. Leisure activities providing indexes for physical, nonphysical hobbies and social activity.<br>4. TUG<br>5. Total number of prescription medications<br>6.1 Mini-Mental State Examination (MMSE)<br>6.2. Digit Span Forward and Backward from Wechsler Adult Intelligence Scale Revised (WAIS-R)<br>6.3 Block Designs-5 blocks, even numbers from the WAIS-R Block Design<br>6.4 Trail-making test A and B	Participants with scores of 2 or 3 were regarded as having vision and/or hearing impairment.<br>Age group differences in the different outcomes using Pearson Chi square and ANOVA.<br>Age group differences in each of the three indexes were examined by t test (nonphysical and social activity indexes) and Wilcoxon rank sum nonparametric test (physical activity index, due to skewed distribution), comparing the youngest age group (65–75 years) with each of two other age groups (75–84 years and 85 years or older).	Pearson chi-square statistics<br>% With either vision or hearing impairment **p<0.001**†<br>IADL disabilities, % None **p<0.001**†<br>% with 21 MMSE **p=0.001**†<br>ANOVA<br>TUG **p<.001**†<br>Total No. of prescription medication **p<0.001**†<br>WAIS-R Digit Span Forward NS<br>WAIS-R Digit Span Backward **p=0.005**†<br>WAIS-R Block Design–5 block designs **p<0.001**†<br>Trail-Making A: connections per second **p<0.001**†<br>Trail-Making B: connections per second **p<0.001**†<br>Nonphysical Activity Index (t test)<br>(65 - 75)-(75-85) p=0,19; (85+)-(65 - 75) **p<0.001**†<br>Physical Activity Index (Wilcoxon rank test)<br>(65 - 75) - (75-85) p=0,45; (85+) - (65 - 75) **p=0.04**†<br>Social Activity Index (t test)<br>(65 - 75) - (75-85) p=0,22; (85+) - (65 - 75) **p<0.001**†

**Pinheiro et al., 2013** [67]<br>(Brazil)	Cross-sectional<br>n=316	74.2 ± 9 .8<br>[60, 105]<br>54.7%	*Objective measures:*<br>1. Handgrip strength (HG) assessed by dynamometry<br>2. Strength/ endurance of the lower limbs assessed by time to perform 5 sit-to-stand<br>3. Mobility/ flexibility assessed by the time to perform a task<br>4. Locomotion assessed by time to walk a path of 2.44 m<br>5. Balance assessed by task performance	1. Hydraulic dynamometer (Saehan Corporation SH5001, Korea)<br>2. Chair stand test (STS)<br>3. Pick-up-a-pen test (PPT) (movement between bending down and returning to the former position)<br>4. Walk test (WT)<br>5. Maintain balance: both feet together, tandem, while standing only on the right/ left leg	The effect of age on motor performance was assessed by the Kruskal-Wallis test 60-69), (70-79), (≥ 80).<br>Chi-square calculations of the age group, sex and categories of test performance (Disabled, Poor, Medium, Good); if any expected frequency was below five, then Fisher's exact test was applied.	Kruskal-Wallis test<br>HG All **p<0.001†**; Men **p<0.001†**; Women **p<0.001†**<br>STS All **p=0.001†**; Men **p<0.001†**; Women **p=0.012†**<br>PPT All **p<0.001†**; Men **p<0.001†**; Women **p<0.001†**<br>WT All **p<0.001†**; Men **p<0.001†**; Women **p<0.001†**<br>Chi-square or Fisher's exact test<br>HG Men **p=0.002†**; Women **p<0.001†**<br>STS Men **p=0.002†**; Women **p=0.009†**<br>PPT Men **p<0.020†**; Women **p=0.001†**<br>WT Men **p<0.020†**; Women **p<0.001†**<br>Balance Men **p<0.001†**; Women **p<0.001†**
**Uttl et al., 2001**[80]<br>(Canada)	Cross-sectional*<br>n=133<br>AG<br>65–69<br>n=31<br>70–74<br>n=41<br>75–79<br>n=38<br>80–95<br>n=23	[65, 95]<br>58.6%	*Self-reported measures:*<br>1. Self-rating about eyesight hearing, and general health<br>*Objective measures:*<br>2. Cognitive function<br>3. Sensation<br>4. Prospective memory (PM) assessed by three brief tasks<br>5. Retrospective memory (RM) assessed by test<br>6. Attention assessed by test<br>7. Semantic fluency assessed by test<br>8. Cognition assessed by test	1. 1=5 excellent to 5=unable to see, 1=excellent to 5=unable to hear and 1=very good to 5=very poor general health<br>2. Modified Mini-Mental State examination (MMS)<br>3. Color Vision Screening Inventory (CVSI)<br>4. The name task, the letter task, and the check task.<br>5. Retrospective Memory tests: Buschke 1-3; RAVLT A1-5; RAVLT A6; RAVLT B1<br>6. Cancel H, Card sorting, Digit symbol<br>7. Verbal fluency, Animal naming, Picture naming<br>8. Modified Mini-Mental State (MMS)	Age effects were computed by regression analysis.	MMS F=23.77, **p<0.05†**; CVSI F=0.04, p>0.05; Vision F= 1.40, p>0.05; Hearing F=1.63, p>0.05; Health F=3.13, p=0.079<br>Tasks PM: Name X^2=14.0, **p=0.001†**; Letter X^2=3.39, p=0.066; Check X^2=5.55, **p=0.019†**<br>Items PM : Name: Pen X^2=9.55, **p<0.05†**; Paper X^2=13.03, **p<0.05†**; Letter: Pen X^2=2.04, p>0.05; Letter X^2=2.53, p>0.05; Envelope X^2=12.21, **p<0.05†**; Check: Pen X^2=4.59, **p<0.05†**; Envelope X^2=11.48, **p<0.05†**; Check X^2=5.16, **p<0.05†**<br>Activities PM: Name: Write X^2=7.58, **p<0.05†**; Letter: Date X^2=5.63, **p<0.05†**; Sign X^2=4.95, **p<0.05†**; Copy name X^2=9.15, **p<0.05†**; Copy street X^2=12.54, **p<0.05†**; Copy city X^2=15.24, **p<0.05†**; Copy postal code X^2=12.90, **p<0.05†**; Check: Date X^2=2.64, p>0.05; Pay to X^2=5.98, **p<0.05†**; Amount X^2=7.84, **p<0.05†**; Amount written X^2=3.75, p>0.05; Sign X^2=2.73, p>0.05; Check in envelop X^2=7.30, **p<0.05†**<br>RM tests: Buschke 1-3 F= 12.32, **p=0.05†**, r^2=0.087; RAVLT A1-5 F=6.26, **p=0.05†**, r^2=0.046; RAVLT A6 F=4.54, **p=0.05†**, r^2=0.034; RAVLT B1 F=7.39, **p=0.05†**, r^2=0.054<br>Attention tests: Cancel H F= 17.14, **p=0.05†**, r^2=0.117; Card sorting F= 42.20, **p=0.05†**, r^2=0.245; Digit symbol F= 18.68, **p=0.05†**, r^2=0.126 Semantic fluency: Verbal fluency F= 8.04, **p=0.05†**, r^2=0.058; Animal naming F=14.79, **p=0.05†**, r^2=0.102; Picture naming F=26.19, **p=0.05†**, r^2=0.168

**Makizako et al., 2017** [60]<br>(Japan)	Cross-sectional<br><br>n=10 092	73.6 ± 5.6<br><br>Male<br>73.7 ± 5.6<br>Female<br>73.6 ± 5.6<br><br>52,5%	*Objective measures:*<br>1. Physical performance assessed by level of grip strength, time to five-time-sit-to-stand (FTSS), and walking speed<br><br>2. Body composition assessed by values of Body weight, Fat mass and Appendicular muscle mass	1. Smedley-type handheld dynamometer (GRIP-D; Takei Ltd, Niigata, Japan), FTSS test and walking speed at 2 meter comfortable pace<br><br>2. Bioelectrical impedance analyzer (MC-980A; Tanita, Tokyo, Japan)	Pearson's correlation coefficients were calculated to assess the simple correlations of physical performance measures and body composition parameters with age.<br><br>To compare the differences in the age-dependent changes among various measures, T-scores were calculated by using the equation: T score= 50+10†(participant's value -population mean)/ population standard deviation. Then, the T-scores for each measure in the 65–69 year age group were set as references (i.e. T-score = 50), and the cumulative mean T-score change for each age group was calculated. For the T-score curve for the FTSS, the curve was inverted (decreasing with advancing age). For the evaluation of the slope decline in age-associated changes among the indicators, authors used linear regression.	Pearson's correlation coefficients<br>Male<br>Grip strength, kg r=-0.44, **p<0.01†**<br>FTSS, s r=0.27, **p<0.01†**<br>Walking speed, m/s r=-0.37, **p<0.01†**<br>Body weight, kg r= -0.23, **p<0.01†**<br>Fat mass, kg r=-0.02, p≥0.01<br>Appendicular muscle mass, kg r=-0.32, **p<0.01†**<br>Female<br>Grip strength, kg r=-0.36, **p<0.01†**<br>FTSS, s r=0.33, **p<0.01†**<br>Walking speed, m/s r=-0.48, **p<0.01†**<br>Body weight, kg r= -0.17, **p<0.01†**<br>Fat mass, kg r=-0.05, **p<0.01†**<br>Appendicular muscle mass, kg r=-0.32, **p<0.01†**<br><br>T-score results confirm that all physical performance measures, BMI and Appendicular muscle mass worsened with advancing age.
**Papadakis et al., 1995** [66]<br>(USA)	Cross-sectional<br><br>n=104	75.5±4.9<br>[70, 94]<br><br>0%	*Objective measures:*<br>1. Muscle strength assessed by Peak torque of knee flexion and extension at joint speeds of 90, 120, and 180 degrees per second (dps) and Grip strength<br><br>2. Physical Performance assessed by time to complete different tasks<br><br>3. Body composition assessed by lean tissue mass and fat mass<br><br>4. Cognitive function assessed by three tests	1. Isokinetic dynamometer (Cybex 340, Lumex Corp., Bay Shore, NY) and grip dynamometer (Smedley Grip Dynamometer, JA Preston, Jackson, MI)<br><br>2. Physical Performance Test (write a prescribed sentence, transfer kidney beans using a teaspoon, place a heavy book on a shelf, remove a jacket, pick up a penny from the floor, turn 360 degrees, walk a 50-foot walk test course, and climb stairs to determine speed and number of flights climbed before the subject fatigued)<br><br>3. Dual-energy X-ray absorptiometry (DEXA) (Lunar DPX-Plus, Madison, WI)<br><br>4. Mini-Mental State Examination, Trails B, and the Digit Symbol Substitution	Multiple linear regression for each variable including age and IGF-1 as independent variables	Muscle strength<br>Knee extension: Peak torque 120 dps (ft-lbs) β=-1.8 **p=0.0001†**, Model r^2=0.174<br>Knee flexion: Peak torque 120 dps (ft-lbs) β=-0.87, **p=0.004†**, Model r^2=0.1 05<br>Handgrip (kg) β=-0.65, **p=0.0002†**, Model r^2=0.19<br><br>Body composition<br>Percent lean tissue mass β=-0.10 p=0.45, Model r^2=0.01<br>Percent fat mass β=- 123.9 p=0.59, Model r^2=0.008<br><br>Physical Performance Test<br>β=-0.27, **p=0.0001†**, Model r2=0.247<br><br>Cognitive function<br>Trails B score β= 3.73, **p=0.0001†**, Model r^2= 0.257<br>Mini-Mental State Exam β=-0.09, **p=0.005†**, Model r^2=0.123<br>Digit Symbol Substitution test β t=-0.85 **p=0.0003†**, Model r^2=0.210

**Romero-Ortuno et al., 2009** [71]<br>(Ireland)	Cross-sectional<br><br>n=355<br><br>AG<br>60–69<br>n = 133<br>70–79<br>n = 153<br>80–89<br>n = 69	72.7 ± 7.2<br><br>AG<br>[60-69]<br>65.3 ± 2.6<br>70-79<br>74.2 ± 2.9<br>[80-89]<br>83.4 ± 2.8<br><br>67.0%	*Objective measures:*<br>1. Preferred walking speed in m/s	1. GAITRite™ walkway system (CIR Systems, Inc., 60 Garlor Drive, Havertown, PA 19083)	Simple linear regression was conducted to investigate if age predicts walking speed<br><br>Backwards multiple linear regression was used to investigate the extent to which age was an independent predictor of walking speed in the presence of other confounders	Linear regression (with 95% confidence interval for the mean) between age and observed walking speed<br>F (1, 353) = 108.48, R^2=0.235, **p<0.001†**<br><br>Backwards multiple linear regression<br>Age (along with other variables)F (4, 329) = 156.23, R^2=0.651, **p=0.001†**
**Tomsone et al., 2013** [78]<br>(Germany, Latvia and Sweden)	Cross-sectional<br><br>n=1098<br><br>Sub-samples<br>German<br>n=419<br>Latvian<br>n=292<br>Swedish<br>n=387	Germany and Sweden sample<br>[80, 89]<br><br>Latvia sample<br>[75, 84]	*Self-reported/ Objective measures:*<br>1. ADL independence/dependence assessed by questionnaire	1. ADL Staircase (combination of interview and observation)	For each of the three national samples differences between the two ADL groups were tested by means of Mann–Whitney's U test for age	Differences between age of ADL groups (dependent/independent)<br>German sample **p<0.0005†**<br>Latvian sample **p<0.0005†**<br>Swedish sample **p<0.0005†**
**Turner et al., 2016** [79]<br>(USA)	Cross-sectional<br><br>n=943	80 ± 7.8<br><br>76%	*Self-reported measures:*<br>1. Sleep quality, sleep apnea assessed by questionnaire	1. 32-item Questionnaire developed by the authors to assess sleep quality and determine the occurrence of sleep apnea and REM behavior disorder, using selected questions from the Pittsburgh Sleep Quality Index, the Berlin Questionnaire, and the Mayo Sleep Questionnaire	Chi-square tests, t-tests and one-way ANOVAs to compare the mean sleep scores across different demographic subgroups (Mean differences for one-way ANOVAs are based on Tukey-Kramer post hoc analysis. Chi-square employed for Sleep apnea risk)	Sleep quality score F3939=2.96, **p=0.031†**<br><br>Sleep apnea risk score X^2=8.41, **p=0.015†**

**Adachi et al., 2015** [33] (Japan)	Cross-sectional<br>n=149	74.0 ± 4.0<br>51%	*Objective measures:*<br>1. Endurance assessed through walked distance	1. Shuttle walking test (SWT)	Comparison of age between higher and lower level of SWT, according t-test.<br><br>Analysis of factors associated with the SWT results using a stepwise multivariate logistic regression model, assigning the high SWT results group as a dependent variable and age as explanatory variable	Female **p<0.001†**<br>Male **p=0.002†**<br><br>Female<br>(Odds Ratio) OR=0.69, Confidence interval 95% CI=0.57–0.82 **p<0.001†**<br>Male<br>p>0.05
**Moreira et al., 2016** [62] (Brazil)	Longitudinal Study (2 years)<br>n=103	76.3 ± 5.95 [67, 92]<br>58.2%	*Self-reported measures:*<br>1. Activities of daily living (ADL)<br><br>2. Instrumental activities of daily living (IADL)	1. Katz Index of ADL<br><br>2. Instrumental activities of daily living by Lawton & Brody, 1969	If individuals gave an affirmative response to at least one of the ADL and IADL questions were defined as presenting difficulties.<br><br>Age comparison between groups (with and without functional decline) were analyzed by Student t-test.<br><br>Multiple logistic regression model was used considering impairment of ADL and IADL as variable response, and several independent variables as age, using a stepwise variable selection method.	Individuals in 2008 that developed or not ADL decline in 2010<br>ADL decline **p=0.04†**<br><br>Study sample in 2008 that developed or not IADL decline in 2010<br>IADL decline **p=0.001†**<br><br>Multiple logistic regression of promoting factors for decline.<br>IADL Age OR 1.12, IC95% 1.02–1.23, **p=0.02†**<br>ADL Age NS
**Nakagawa et al., 2017** [63] (Brazil)	Cross-sectional<br>n=202	Female<br>70.2 ± 5.6<br>Male<br>71.1 ± 6.9<br>57.9%	*Objective measures:*<br>1. Functional balance assessed by activity performance scale<br><br>*Self-reported measures:*<br>2. Functional independence in daily life assessed by scale	1. Berg Balance Scale (BBS)<br><br>2. Barthel Index (BI)	Comparisons between the age groups (60-69), (70-79), (≥ 80) were made using oneway ANOVA, with Dunn's post-test.<br><br>The effect of independent variables on the dependent variable (Berg or Barthel), was calculated by a multiple linear regression model, constructed by means of the Enter method (forced input). R^2 was analyzed to ascertain the coefficient of determination of the percentage variation explained by the model.	Comparisons between age groups<br>BBS p=0.000†<br>Dunn's post-test<br>(60-69) vs (70-79), **p<0.01†**<br>(60-69) vs (≥ 80), **p<0.001†**<br>(70-79) vs (≥ 80), **p<0.001†**<br>BI p=0.205<br><br>Multiple linear regression<br>BBS: Model R^2=0.369, Age β(95% CI)=-0.344 (-0.424, -0.265), **p=0.0001†**<br>BI: Model R^2=0.122, Age β(95% CI)=--0.086 (-0.141, -0.030), **p=0.003†**

**Sarvestan et al., 2021** [73]<br>(Czech Republic)	Cross-sectional*<br><br>n=27	69.1±5.7<br><br>100%	*Objective measures:*<br>1. Gait and balance assessed by test<br>2. Gait speed assessed by test<br>3. Balance assessed by sway velocity in Anteroposterior (AP), Mediolateral (ML) and Vertical (V) directions in standing position during 30 seconds<br>4. Strength of Ankle invertors (AIN) and evertors (AEV), Ankle plantar flexors (APF), Ankle dorsi flexors (ADF), Knee flexors (KFL) and extensors (KEX), Hip abductors (HAB) and adductors (HAD), Hip flexors (HFL) and extensors (HEX) assessed by dynamometry	1. Performance Oriented Mobility Assessment (POMA)<br>2. The six-minute walk (SMW) test<br>3. AMTI force platform (Model OR6-5, Newton, Watertown, MA, USA; sampling frequency 200 Hz)<br>4. Iso-Med 2000 Isokinetic dynamometer (D&RFerstl, Hemnau, Germany)	The correlation between age with walking performance variables, functional mobility tests and balance performance was investigated using the Pearson's product-moment correlation coefficient	POMA r=-0.51, **p=0.01†**<br>Average walking speed (m.s-1) r=0.62, **p<0.01†**<br>Sway velocity (mm.s-1)<br>AP r=0.55, **p<0.01†**<br>ML r=0.18, p=0.59<br>V r=0.54, **p<0.01†**<br>AIN (Nm.kg-1) r=-0.49, **p=0.01†**<br>AEV Nm.kg-1) r=-0.43, **p=0.04†**<br>APF (Nm.kg-1)r=-0.65, **p<0.01†**<br>ADF (Nm.kg-1) r=-0.13, p=0.69<br>KFL (Nm.kg-1) r=-0.50, **p=0.01†**<br>KEX (Nm.kg-1) r=-0.47, **p=0.03†**<br>HAB Nm.kg-1) r=-0.62, **p<0.01†**<br>HAD (Nm.kg-1) r=-0.37, p=0.10<br>HFL (Nm.kg-1) r=-0.53, **p<0.01†**<br>HEX (Nm.kg-1) r=-0.57, **p<0.01†**
**Tomita & Burns, 2013** [77]<br>(South Africa)	Cross-sectional<br><br>n=1429	≥65<br><br>62%	*Self-reported measures:*<br>1. Functional status assessed by the areas, activities of daily living (ADL), instrumental activities of daily living (IADL), and physical functioning and mobility (PFM)	1. ADL: "Please indicate the level of difficulty you have with each activity...in dressing, bathing, eating and toileting by yourself"; IADL: "Please indicate the level of difficulty you have with each activity...in taking a bus, taxi and train by yourself, doing light work in or around the house, managing money, and cooking for yourself"; PFM: "Please indicate the level of difficulty you have with each activity...in climbing a flight of stairs, lifting and carrying heavy objects (e.g. a bag weighing 5 kg), and walking 200–300 meters"	Functional status in ADL, IADL, and PFM was classified as two levels: 'difficulty' and 'dependence'.<br>Differences in the functional status by different age groups were assessed using either t-test or Pearson chi-square statistics, adjusted using the poststratification weight, with the second-order correction method for survey design and eventually converted into F statistics.	Functional status across age groups among 65+ age group<br>ADL difficulty F(1.85, 640.57) = 2.65,p=0.08<br>IADL difficulty F(1.68, 584.34) = 4.45, **p=0.02†**<br>PFM difficulty F(1.51, 524.81) = 5.45, **p=0.01†**<br>ADL dependence F(1.49, 515.78) = 2.62, p=0.09<br>ADL dependence (>85+ vs. (65–84) F = 7.10, **p<0.01†**<br>IADL dependence F(1.83, 506.56) = 9.54, **p<0.01†**<br>PFM dependence F(1.57, 433.66) = 7.01, **p<0.01†**
**Zunzunegui et al., 2006** [81]<br>(Spain)	Longitudinal Study<br>(6 years)<br><br>n=1283	≥65<br><br>49.4%	*Self-reported measures:*<br>1. ADL assessed by questionnaire<br>2. Functional limitations assessed by questionnaire<br>3. Health status assessed by Self-rated health (SRH)	1. Index of basic physical activities - Nagi<br>2. No difficulty, slight difficulty, great difficulty and unable to do it in stooping/kneeling, reaching/extending arms, pulling/pushing large objects like chairs, and handling or picking up small objects. These questions were combined into two categories to distinguish those who had great difficulty or were unable to do any of the four activities.<br>3. "How would you rate your health?"	Logistic regression with fixed effects (i.e., the relation between outcome variable and explanatory variables is the same for every subject). Three series of analysis were carried out separately, to estimate the probability of ADL disability, functional limitations and poor self-rated health.	Logistic regression coefficient to estimate population average estimates of ADL disability prevalence.<br>Age Coefficient=0.123 SD=0.014, p>0.05<br><br>Logistic regression coefficient to estimate population average estimates of prevalence of functional limitations.<br>Age Coefficient=0.091, SD=0.007, p>0.05<br><br>Logistic regression coefficients to estimate population average estimates of prevalence of poor self-rated health.<br>Age Coefficient=-0.001, SD=0.007, p>0.05

**Barberger et al., 1992** [39]<br>(France)	Cross-sectional<br><br>n=2792	75.0 ± 7.0<br><br>59,9%	*Self-reported measures:*<br>1. Health status assessed by questionnaires of ADL, IADL, Mobility and Global self-perceived health<br><br>2. Physical health assessed by the presence of visual or hearing impairments and two symptoms (dyspnea and joint pain)<br><br>*Objective measure:*<br>3. Mental health assessed by cognitive test	1.1 Katz Index of Independence in ADL<br>1.2 Lawton And Brody IADL Scale<br>1.3 Index of Mobility scale (Rosow and Breslau)<br>1.4 Mobility assessed by a scale: bound to bed, home, neighborhood, district, plain difficulties in using means of transportation, no restriction in mobility<br>1.5 Global self-perceived: Subjective health: "How would you rate your health status presently: very good? good? fair? bad? very bad?"; Relative health: "In comparison to other people of the same age, do you feel rather: better, the same, or worse?"<br>2.1 "Do you feel some problems when listening to a conversation in a group or in a noisy environment?"<br>2.2 "Do you suffer from visual problems that impair activities such as reading or sewing?" even if you are wearing a hearing aid or glasses. 2.3 "Do you feel out of breath in some of the following circumstances: at rest, in performing ADLs, for minor efforts walking at the same pace as other people of the same age, for major efforts climbing more than one flight of stairs, never?"<br>2.4 "Do you suffer from pain in your joints?"<br>3. Mini Mental State Exam (MMSE)	Associations Between Age and the health measures, test non reported<br><br>Associations between the subject's age and the six health measures (ADL, IADL, Mobility, Rosow, Subjective Health, Relative Health) were studied. Bivariate analyses were first performed, using t-test for age.<br><br>A logistic regression was performed for each of the six scales, classifying the subjects into the "dependent" or "independent" group. Odds ratios (ORs) were estimated for a difference of one unit (one year) (Subjects were classified as "dependent" for ADL if they were dependent for at least one of the six activities, for IADL if they were unable to perform one of the five (for men) or eight (for women) tasks of Lawton's scale, for mobility if they were restricted to their bed or their house, for the Rosow-Breslau scale was defined by at least one "No" answer in the three items, for subjective health if they rated themselves in "bad or very bad" health, for relative health those answering they felt "worse" than people of the same age.	Associations Between Age and the health measures<br>Joint pain **p<0.001†**<br>Dyspnea **p<0.001†**<br>Visual impairment **p<0.001†**<br>Hearing impairment **p<0 .001†**<br>MMSE score p>0.05<br><br>Associations Between Dependency and Age<br>ADL **p<0.001†**<br>IADL **p<0.001†**<br>Mobility **p<0.001†**<br>Rosow **p<0.001†**<br>Subjective Health **p<0.001†**<br>Relative Health **p<0.05†**<br><br>Correlations of Being "Dependent" With Age<br>ADL OR= 1.06; **p<0.001†**<br>IADL Male OR= 1.10; **p<0 .001†**<br>IADL Female OR=1.13; **p<0.001†**<br>Mobility OR=1.10; **p<0.001†**<br>Rosow OR=1.13; **p<0.001†**<br>Subjective health OR=0.99; ns<br>Relative health OR=0.95; **p<0.001†**
**Chung et al., 2016** [44]<br>(Hong Kong)	Cross-sectional<br><br>n=944<br><br>AG<br>(65–69)<br>n=248<br>(70–74)<br>n=224<br>(75–79)<br>n=251<br>(80–84)<br>n=221	Male<br>74.3±5.76<br><br>Female<br>74.3±5.61<br><br>55.3%	*Objective measures:*<br>1. Functional fitness assessed by seven testing items<br><br>2. Forearm strength assessed by the highest-rated grip performance<br><br>3. Static balance assessed by the time to became unbalanced	1. Senior Fitness Test (SFT) battery: Body mass index (BMI) 30-s arm curl test (AC), 30-s chair stand test (CS), the back scratch test (BS), chair sit-and-reach test (CSR), the 8-foot up-and-go test (UG) and the 2-min step test (Step)<br><br>2. Hand grip test (HG)<br><br>3. Single leg stance test (SLS)	ANOVA performed to determine the AG effect on each testing parameter for men and women separately. A planned contrast was used to explore the differences between any two adjacent AGs. Because three comparisons were included, the significant p value was adjusted to 0.017. a = difference between (65–69) and (70–74), b = differences between (70–74) and (75–79), c = difference between (75–79) and (80–84)	BMI: Men a; b; c NS; Women a; b; c NS;<br>BS: Men a NS; b; c **p<0.017†**; Women a; b NS; c **p<0.017†**<br>CSR: Men a NS; b; c **p<0.017†**; Women a NS; b; c **p<0.017†**<br>UG: Men a; b; c **p<0.017†**; Women a NS; b; c **p<0.017†**<br>CS: Men a; b; c **p<0.017†**; Women a; b NS; b; c **p<0.017†**<br>AC: Men a; b; c **p<0.017†**; Women a; b; c NS;<br>Step: Men a NS; b; c **p<0.017†**; Women a; b NS; c **p<0.017†**<br>HG Men a NS; b; c **p<0.017†**; Women a; b; c **p<0.017†**<br>SLS eyes open: Men a; b; c **p<0.017†**; Women a; b; c **p<0.017†**<br>SLS eyes closed: Men a; b NS; c **p<0.017†**; Women a; b NS; c **p<0.017†**

**Dong et al., 2014** [48]<br>(USA)	Cross-sectional<br><br>n=3159	72.8±8.3<br>[60, 105]<br><br>58.9%	*Self-reported measures:*<br>1. Physical function assessed with four measures of ability to perform common daily activities<br><br>2. Overall health status (OHS) assessed by question<br><br>3. Health changes over the last year (HC) assessed by question<br><br>*Objective measures:*<br>4. Physical function assessed with physical performance tests	1.1. Katz Index of activities of daily living (ADL)<br>1.2. Lawton instrumental ADL (IADL)<br>1.3 Index of mobility (Rosow and Breslau (ROS))<br>1.4. Index of basic physical activities - Nagi<br><br>2. “In general, how would you rate your health?” on a four-point scale.<br><br>3. “Compared to one year ago, how would you rate your health now?” on a three-point scale.<br><br>4.1. Chair stand<br>4.2. Tandem stand<br>4.3. 8-foot timed walk	Pearson and Spearmen correlation coefficients were calculated to determine the relationships between age and health-related variables and composite score of each physical function test.	ADL r=0.27, **p<0.001†**<br>IADL r=0.48, **p<0.001†**<br>ADLIADL r=0.48, **p<0.001†**<br>NAGI r=0.38, **p<0.001†**<br>ROS r=0.44, **p<0.001†**<br>OHS r=-0.08, **p<0.001†**<br>HC r=-0.11, **p<0.001†**<br>Chair r=-0.34, **p<0.001†**<br>Tandem r=-0.40, **p<0.001†**<br>Walk r=-0.38, **p<0.001†**<br>PhyP (physical performance tests summary score)r=-0.46, **p<0.001†**
**Furuna et al., 1998** [51]<br>(Japan)	Longitudinal Study<br>(4 years)<br><br>n=517<br><br>AG<br>(65–69)<br>n = 252<br>(70–74)<br>n = 159<br>(75–79)<br>n = 73<br>(>80)<br>n = 33	NR<br><br>62.5%	*Objective measures:*<br>1. Muscle Strength assessed by Grip Strength in Kg<br><br>2. Balance assessed by the time (seconds) in one-leg Standing with or without vision<br><br>3. Walking speeds assessed by velocity (m/s) of walking at preferred and maximum speeds<br><br>4. Manual speed assessed by maximum finger-tapping rate (Hz)<br><br>*Self-reported measures:*<br>5. Functional capacity assessed by IADL index	1. Dynamometer<br><br>2. One-leg Standing with or without vision using the preferred leg<br><br>3. Walk test (11 meters)<br><br>4. Tapping the middle finger (metal knob) against a stainless-steel plate. The metal knob and the plate formed an electrical circuit.<br><br>5.TMIG Index of Competence	To detect longitudinal changes in physical performance, a repeated-measure ANOVA with the age-group and double replication of measurement serving as factors was applied to each physical performance.<br><br>A logistic multiple regression analysis was performed to predict ability to maintain the level of IADL for next 4 years by the baseline age. IALD was coded as "1" when the total score for Instrumental Self-Maintenance in the TMIG Index of Competence decreased in the 4 year follow-up examination, and "0" if the score maintained or increased. Deterioration of IADL was assumed to be a dependent variable. Age was assumed to be independent variable.	The cross-sectional decline was also evident in all physical performances F>9.1, **p<0.0001†**<br><br>Significant interaction between age-group and longitudinal change in preferred walking speed F=3.3, **p<0.05†**<br><br>Logistic regression Analysis IADL<br>Age Wald=14.8, OR 1.10, CI95%=1.05-1.15, **p<0.01†**

**Grassi et al., 2020** [53] (Italy, Germany, Spain, Switzerland, UK, Israel)	Cross-sectional<br><br>n=3142	73.7 ± 5.6<br><br>50.7%	*Self-reported measures:*<br>1. Functioning assessed by questionnaire<br><br>2. Self-Health Rated (SHR) assessed by single question	1. World Health Organization Disability Assessment Schedule II (WHODAS-II)<br><br>2. How do you rate your overall health in the past 30 days?, a score of 1 corresponding to “very good” and 5 to “very bad”	Linear regressions were used to analyze the effect of four age groups (65–69, 70–74, 75–79, > 80) on the WHODAS-II, its subscales and SHR.	WHODAS II total score **p<0.001†**<br>Mobility **p<0.001†**<br>Household **p<0.001†**<br>Cognitive **p<0.001†**<br>Social p=0.426<br>Self-care **p<0.001†**<br>Society **p<0.001†**<br><br>SHR **p<0.05†**
**Jansen et al, 2008** [59] (USA)	Cross-sectional<br><br>n=224	75.4 ± 6.8 [65, 92]<br><br>62.5%	*Objective measures:*<br>1. Hand strength assessed by grip-force, tip pinch force, key pinch force and three jaw-chuck pinch force	1. Jamar dynamometer and B&L Engineering pinch gauges	ANOVA was used to analyze differences of grip and pinch force among five age groups (65–69 years, 70–74 years, 75–79 years, 80–84 years, and 85+ years).	Grip Force<br>age group F=28.72, **p=0.01†**<br>Key Pinch Force<br>age group F=17.02, **p=0.01†**<br>Three-Jaw-Chuck Pinch Force<br>age group F=15.42, **p=0.01†**<br>Tip Pinch<br>age group F=6.55, **p=0.01†**
**Prata & Scheicher, 2012** [70] (Brazil)	Cross-sectional<br><br>n=70	70.5±5.0<br><br>81.4%	*Objective measures:*<br>1. Balance assessed by a scale<br>2. Cognitive impairment assessed by test<br>*Self-reported measures:*<br>3. Functional independence in ADL evaluated by an index	1. Berg Balance Scale (BBS)<br>2. Mini-Mental State Examination (MMSE)<br><br>3. Barthel Index (BI)	The Spearman correlation coefficient was used to examine the relationship between the evaluation parameters.	Association between age and BBS r=-0.57, **p=0.0001†**<br>Association between age and MMSE r=0.34, **p=0.0032†**<br>Association between and BI r=-0.24, **p=0.04†**
