# Supplementary material for: Indicators and Instruments to Assess Components of Disability in Community-Dwelling Older Adults: A Systematic Review
Source: Sensors (Basel). 2022 Oct 28;22(21):8270. doi: 10.3390/s22218270 (PMC9653663; doi:10.3390/s22218270)
Supplement: Supplementary file 1 [file sensors-22-08270-s001.zip › Table_S2.pdf]

Table S2. Characterization and summary of studies included in the review with classification of “Fair” in Downs and Black scale, listed by decreasing order of quality score (for those with same score alphabetic order was used).

Notes: AG – age group, ANOVA - Analysis of variance; M±SD – Mean plus Standard Deviation; NR – non reported; NS – non significant, * - study design non reported in the respective study, and thus classified by the reviewers; † - indicators with significative change or association with age.

ID	**Study Design** (Follow-up time) **Sample Size**	**Age** M±SD [range] % female	**Indicators** Objective & Self-reported	**Instrument**	**Main statistical strategy used to assess the influence of age**	**Significative change/ association with age**
**Alcock et al., 2015** [34] (United Kingdom)	Cross-sectional<br><br>n=39	71.5 ± 7.3<br>[60, 83]<br><br>100%	*Objective measures:*<br>1. Gait speed assessed by central part of 10 meter walk<br>2. Time to complete TUG<br>3. Time to compete one sit-to-stand cycle<br>4. Knee flexor and extensor concentric strength assessed by Hamstrings-to-Quadriceps ratio, Peak Knee Extensor and Flexor Moment (PKnee EM, PKnee FM)<br>*Self-reported measures:*<br>5. Health and well-being status assessed by questionnaire	1. 10-meter walk<br>2. TUG test<br>3. Optoelectronic system<br>4. Dynamometer<br>5. Short Form Health Survey (SF-36)	Bivariate correlations (Pearson’s r, R2 and significance level) expressed the strength of the relationship between each of the outcomes and age.<br><br>Linear regression was computed for all outcomes using age as a single independent predictor (regression coefficient beta (β), significance level).	Gait speed (m/s) r=-0,57, R^2=32%, **p≤0.01†**; β=-0.017, **p≤0,001†**<br>TUG time(s) r=0.53, R^2=28%, **p≤0.01†**; β=0.181, **p=0.001†**<br>STS time (s) r =0,15, R^2=0%, p>0.05; β=-0.085, p=0.386<br>HQ ratio (%) r=-0.46, R^2=21%, **p≤0.01†**; β=-0.010, **p≤0,001†**<br>PKnee EM(Nm/kg) r=-0.58, R^2=33%, **p≤0.01†**; β=-0.014, **p≤0.001†**<br>PKnee FM(Nm/kg) r=-0.58, R^2=34%, **p≤0.01†**; β=-0.009, **p≤0.001†**<br>SF-36:<br>Physical Functioning r=-0.53, R^2=28%, **p≤0.01†**; β=-1.143, **p≤0.001†**<br>Role-Physical r=-0.19, p=not computed due to ceiling effects<br>Bodily Pain r=-0.30; R^2=9%, p>0.05; β=0.835, p=0.065<br>General Health r=-0.21; R^2=10%; p>0.05; β=-0.685, p=-0.059<br>Vitality r=-0.29; R^2=9%, p>0.05; β=-0.770, p=0,074<br>Social Functioning r=-0.03, p=not computed due to ceiling effects<br>Role- Emotional r= -0,11, p= not computed due to ceiling effects<br>Mental Health r=-0.06, R^2=0%, p>0.05; β=-0.105, p=-0.701<br>Mental Component r=0,01, R^2=0%, p>0.05; β=0.008, p=0.958<br>Physical Component r=-0,38, R^2=, **p≤0.01†**; β=-0.443, **p=0.022†**
**Amarasena et al., 2018** [35] (Australia)	Cross-sectional<br><br>n=354<br><br>AG<br>(75-84)<br>n=274<br>(≥85)<br>n=80	NR<br><br>52%	*Self-reported measures:*<br>1. Self-rated oral health (SROH) assessed trough single question<br>2. Self-rated General health (SRGH) assessed trough single question	1. How would you rate the overall health of your teeth, dentures and gums?<br>2. ‘In general, would you say your health is. . .?’.	SRGH and SROH responses dichotomized into categories ‘poor/fair’, ‘good/very good/excellent’ were cross-tabulated with age [AG 75–84, ≥85 years)] using chi-squared test.<br><br>Adjusted analyses performed using log-binomial regression.	Good SROH<br>(75–84) (37.3%), p≥0.05<br>(≥85) 29 (37.2%), p≥0.05<br>Good SRGH<br>(75-84) n=153 (56.7%), p≥0.05<br>(≥85) n=50 (63.3%), p≥0.05<br><br>Good SROH: Prevalence ratio=0.90 (95% CI 0.64–1.27), p≥0.05<br>Good SRGH: Prevalence ratio=1.03 (95% CI 0.85–1.25), p≥0.05

**Arroyo et al., 2007** [38]<br>(Chile)	Cross-sectional<br><br>n=377	Male<br>73,9 ± 6,1<br>[65, 91]<br><br>Female<br>74,4 ± 6,6<br>[65, 95]<br><br>63,1%	*Self-reported measures:*<br>1. Functional ability assessed by questionnaire	1.1. ADL, IADL questionnaire (scale non-identified)<br><br>1.2. AADL (advanced activities of daily life) questionnaire including items of Rosow and Breslau and Index of basic physical activities – Nagi	Author defined the report of difficulties in at least 1 ADL or 1 AIVD or 2 AAVD to define the functional limitation.<br>Multiple logistic regressions performed to study the association of functionality and mobility with age.	Multiple Logistic regression<br>Male<br>Age OR=1,04, IC 95%=0,975-1,117, p=0,122<br>Female<br>Age OR=1,098, IC 95%=1.043-1.155, **p<0.001†**
**Chang & Dong, 2014** [40]<br>(USA)	Cross-sectional*<br><br>n=3159	72.8 ± 8.3<br>[60, 105]<br><br>58.9%	*Objective measure:*<br>1. Cognitive function assessed by a battery of five cognitive function test	1.1. Global cognition: Chinese Mini-Mental State Exam (C-MMSE)<br><br>1.2. Episodic memory: summarized scores of immediate recall (East Boston Memory Test (EBMT) -Immediate Recall and delayed recall of brief stories in the EBMT (range 0–24)<br><br>1.3. Executive function: 11-item Symbol Digit Modalities Test (SDMT)<br><br>1.4. Working memory: Digit Backwards (DB) from Wechsler Memory Scale-Revised	A composite score (scale name+Z) for global cognitive function was calculated by transforming a participant's score on each cognitive test to a z score based on the M and SD of the distribution of the scores of all participants on that test, and then averaging z scores across tests.<br><br>Spearman correlation coefficients were calculated to determine relationship between age and cognitive function. Based on the results of bivariate analysis, was performed multivariate analysis using linear regression models to report the effect of age and health-related variables on composite measure of function by cognitive domain.	Spearman Correlations Between Composite Scores of Cognitive Function Domains and Age<br>GlobCog r=−0.33, **p<0.001†**<br>C-MMSE Z r=−0.31, **p<0.001†**<br>Episodic memory r=−0.28, **p<0.001†**<br>SDMT Z r=−0.35, **p<0.001†**<br>DB Z r=−0.22, **p<0.001†**<br><br>Effect of age on composite measure of function by cognitive domain: Estimated Slope (SE)<br>Global cognitive score −0.030 (0.002), **p<0.001†**<br>SDMT Perceptual speed −0.034 (0.002), **p<0.001†**<br>Episodic memory −0.031 (0.002), **p<0.001†**<br>DB −0.020 (0.002), **p<0.001†**<br>C-MMSE Z score −0.038 (0.002), **p<0.001†**
**Chen et al., 2012** [42]<br>(Taiwan)	Cross-sectional*<br><br>n=384<br><br>Young-old<br>(65-74)<br>n=128<br>Middle-old<br>(75-84)<br>n=127<br>Old-old<br>(≥85)<br>n=129	79.2 ± 8.5<br>[65, 101]<br><br>49.0%	*Self-reported measures:*<br>1. ADL assessed by index and ability to complete 5 tasks independently<br><br>2. Physical Status assessed by report of chronic illness conditions<br><br>*Objective measures:*<br>3. Physical Status assessed by cardiovascular-respiratory functions and body composition	1. Barthel index (BI) and Likert-type scale for the tasks: ability to walk outside, shop, take public transportation, do housework, ad manage money<br><br>2. Number of chronic diseases, frequency of doctor's visit, frequency of hospitalization<br><br>3. Respiration rate (times/min), Pulse rate (times/min), Breath-holding duration (s), Lung capacity (L) (TruZone Peak FlowMeter), Systolic and Diastolic blood pressure (mmHg) (sphygmomanometer (OMRON-HEM707), Body height (cm) and weight (kg), Body mass index, Body fat (%) (digital body fat scale TANITA-TBF521), Waist circumference (cm)	Analysis of variance and nonparametric Kruskal-Wallis analysis of variance were conducted to compare the three age groups. Post hoc Bonferroni and Mann-Whitney U tests were performed afterward on differences identified as significant.	Activities of daily living: BI F=4.2, **p=0.015†**, Y> O; Ability to walk outside X^2=50.0, **p<0.001†**, Y>O, M>O; Ability to shop X^2=65.4, **p<0.001†**, Y>M>O; Ability to take public transportation X^2=73.8, **p<0.001†**, Y>M>O; Ability to do housework X^2=37.6, **p<0.001†**, Y>M>O; Ability to manage money X^2=31.0, **p<0.001†**, Y>M>O<br>Chronic illness conditions: Nº of chronic diseases F=0.2, p=0.787; Frequency of doctor's visit F=0.7, p=0.516; Frequency of hospitalization F=0.1, p=0.927<br>Cardiovascular-respiratory functions: Respiration rate F=4.3, **p=0.014†**, Y<O; Pulse rate F=0.9, p=0.393; Breath-holding duration F=11.1, **p<0.001†**, Y>M, Y>O; Lung capacity F = 29.4, **p<0.001†**, Y>M>O; Systolic blood pressure F = 1.9, p=0.146; Diastolic blood pressure F = 24.6, **p<0.001†**, Y>O, M>O<br>Body composition: Body height F=3.4, **p=0.036†**, Y>O; Body weight F=19.7, **p<0.001†**, Y>O, M>O; Body mass index F=11.6, **p<0.001†**, Y>O, M>O; Body fat F = 10.0, **p<0.001†**, Y>O, M>O; Waist circumference F = 2.0, p=0.141

**Ghinescu et al., 2014** [52] (Romania)	Cross-sectional<br>n=547	71.64±5.33<br>58%	*Self-reported measures:*<br>1. ADL and IADL assessed by a scale<br>2. Self-rated health assessed by one question<br>3. Morbidity assessed by chronic conditions scale<br>4. Functioning assessed by questionnaire	1. Groningen Activity Restriction Scale<br>2. Respondents evaluated their health from poor, average, good, very good or excellent (1 to 5, respectively)<br>3. Report the number of chronic conditions in the past 12 months<br>4. Sort Form (SF)-20	Indicators relationship analyzed by factor analysis (principal component, varimax rotation). Factor scores per respondent on each component were calculated. Association of the socio-demographic data, psychological characteristics and social support with dimensions that resulted from factor analysis was analyzed using stepwise linear regression analysis.	By principal component analysis it was identified 3 factors: Factor 1 - denominated independent functioning; Factor 2 denominated suffering from chronic diseases; Factor 3 denominated psychological health<br>Factor 1 Independent functioning: $\beta = -0.321$, **p<0.01†**<br>Factor 2 Suffering from chronic diseases: $\beta = 0.101$, **p<0.05†**<br>Factor 3 Psychological health: $\beta = -0.082$, **p<0.05†**
**Hershman et al., 1995** [56] (USA)	Longitudinal Study (2 to 10 years)<br>n=488	79.2 ± 3.1 [75, 85]<br>64.5%	*Objective and Self-reported measure:*<br>1. Prescription and nonprescription drug use queried during an interview with a nurse	1. Medication type and dose were ascertained by examination of prescription bottles and/or prior medical records.	Differences between means were analyzed using independent t tests. Differences between medication classes were analyzed using Chi square tests.	Medication Use as a Function of Age and Sex<br>Male: Total use <80 vs >80, **p<0.05†**; Prescription use <80 vs >80, **p<0.05†**; Nonprescription use <80 vs >80, p≥0.05<br>Female: Total use <80 vs >80, p≥0.05; Prescription use <80 vs >80, p≥0.05; Nonprescription use <80 vs >80, p≥0.05<br>Frequency of Medication Use by Age ≤80 vs >80<br>Vitamins p=0.52; Diuretics p=0.60; NSAIDS p=0.96; GI p=0.20; Analgesics p=0.24; Sedatives p=0.08; Digoxin p=0.52; Nitrates p=0.27; Beta Blockers p=0.71; Antihistamines X2=10.5, **p=0.001†**; Eye p=0.49; Hypoglycemics p=0.14; Antiarrhythrnics p=0.98; Antidepressants X2 = 4.5, **p=0.03†**; Antibiotics p=0.38
**Ignasiak et al., 2020** [57] (Poland)	Cross-sectional<br>n=5367	69,63±7,06 [60, 93]<br>Male 69,91±6,86<br>Female 69,55±7,11<br>77.6%	*Objective measure:*<br>1. Functional fitness assessed by six tests<br>2. Body composition assessed by Body mass index	1. Senior Fitness Test including 30-Second Chair Stand Test (STS) (perform as many correct lifts from the chair as possible within 30s); Arm curl test (AC) (perform as many correct forearm bends as possible within 30s); Chair Sit and Reach test (CSR) (distance from the middle finger to the toes is measured); Back Scratch test (BS) (distance between the middle fingers of both hands is measured); 8-Foot Up-and-Go Test (8UG) (time needed to complete the task) and 6-Minute Walk Test (6MWT) (distance covered by the examined person in the given time)<br>2. Height and weight assessed by SECA measuring device	Differences between means in the groups were analyzed using two-factor analysis of variance ANOVA with NIR post hoc test. The F statistic was counted for main effects, and the significance of inter-group differences was assessed.	ANOVA: BMI F=2.22, **p=0.0499†**; STS F=34.44; AC F=75.41; CSR F=13.58; BS F=33.39; 8UG F=69.32; 6MWT F=85.73, all **p=0.0000†**<br>NIR post hoc test for comparisons of male and female age groups<br>Male: STS (60–64)vs(70–74) p=0.9263; (65–69)vs(75–79) **p=0.0020†**; (70–74)vs(80–84) **p=0.0003†**; (75–79)vs(85+) **p=0.0000†**; AC (60–64)vs(70–74); (65–69)vs(75–79); (70–74)vs(80–84) **p=0.0000†**; (75–79)vs(85+) **p=0.0001†**; CSR (60–64)vs(70–74) **p=0.0020†**; (65–69)vs(75–79) p=0.9380; (70–74)vs(80–84) p=0.6136; (75–79)vs(85+) **p=0.0094†**; BS (60–64)vs(70–74) **p=0.0177†**; (65–69)vs(75–79) **p=0.0020†**; (70–74)vs(80–84) **p=0.0101†**; (75–79)vs(85+) **p=0.0000†**; 8UG (60–64)vs(70–74) p=0.0719; (65–69)vs(75–79); (70-74)vs(80–84) **p=0.0000†**; (75–79)vs(85+) **p=0.0077†**; 6MWT (60–64)vs(70–74) **p=0.0003†**; (65–69)vs(75–79); (70–74) vs(80–84); (75–79)vs(85+) **p=0.0000†**<br>Female: STS (60–64)vs(70–74); (65–69)vs(75–79); (70–74)vs(80–84); (75–79)vs(85+) **p=0.0000†**; AC (60–64)vs(70–74); (65–69) vs(75–79); (70–74)vs(80–84); (75–79)vs(85+) **p=0.0000†**; CSR (60–64) vs(70–74), (75–79) vs (85+) **p=0.0000†**; (65–69)vs(75–79) **p=0.0003†**; (70–74) vs (80–84) p=0.1917;; BS (60–64) vs (70–74) **p=0.0009†**; (70–74) vs (80–84) **p=0.0005†**; (65–69) vs (75–79, (75–79) vs (85+) **p=0.0000†**; 8UG (60–64) vs (70–74); (65–69) vs (75–79); (70–74)vs(80–84); (75–79)vs(85+) **p=0.0000†**; 6MWT (60–64) vs (70–74); (65–69) vs (75–79); (70–74) vs (80–84); (75–79) vs (85+) p=**0.0000†**

**Marques et al., 2014** [61] (Portugal)	Cross-sectional<br><br>n=4712	75.0 ± 7.2<br>[65, 103]<br><br>66.2%	*Objective measures:*<br>1. Functional fitness assessed by six tests<br><br>2. Anthropometric Measures assessed by BMI level (kg/m^2) and size of waist circumference (cm)	1. Senior Fitness Test which includes 30-Second Chair Stand Test (STS) (perform as many correct lifts from the chair as possible within 30s); Arm curl test (AC) (perform as many correct forearm bends as possible within 30s); Chair Sit and Reach test (CSR) (distance from the middle finger to the toes is measured in cm); Back Scratch test (BS) (distance between the middle fingers of both hands is measured in cm); 8-Foot Up-and-Go Test (time needed to complete the task in seconds) and 6 – Minute Walk Test (6MWT) (distance in meters covered by the examined person in the given time)<br><br>2. Portable stadiometer and balance weighing scales for BMI and heavy-duty inelastic tape for waist circumference	A two-way ANOVA was used to test for main effects and interaction effects across gender and age groups (65–69, 70–74, 75–79, 80–84, and ≥85 years). Bonferroni post hoc tests were then used when warranted (p<0.05).	STS: Male **p<0.001†**: (70-74)vs(65–69), (75-79)vs(65–69) and (70–74), (80-84)vs(65–69), (70–74) and (75-79), (≥85)vs(65–69), (70–74) and (75-79); Female **p<0.001†**: (75-79) vs (65–69) and (70–74), (80–84) vs (65–69), (70-74) and (75–79), (≥85) vs all AG<br>AC: Male **p<0.001†**, (75-79) vs (65–69) and (70–74), (80-84)vs (65–69), (70–74) and (75-79), (≥85)vs(65–69), (70–74) and (75-79)<br>Female **p<0.001†**, (75-79)vs(65–69) and (70–74), (80-84)vs(65-69), (70–74) and (75-79), (≥85) vs (65–69), (70–74) and (75-79)<br>6MWT: Male **p<0.001†**, (70-74)vs(65–69), (75-79)vs(65–69) and (70–74), (80-84)vs(65–69), (70–74) and (75-79), (≥85) vs all AG; Female **p<0.001†**, (70-74)vs(65–69), (75-79)vs(65–69) and (70–74), (80-84)vs(65–69), (70–74) and (75-79), (≥85) vs all AG<br>CSR: Male **p<0.001†**, (75-79)vs(65-69), (80-84)vs(65–69), (70–74) and (75-79), (≥85) vs (65–69), (70–74) and (75-79); Female **p<0.001†**, (75-79) vs (65–69) and (70–74), (80-84) vs (65–69), (70–74) and (75-79), (≥85)vs(65–69), (70–74) and (75-79)<br>BS: Male **p<0.001†**, (70-74) vs (65-69), (75-79)vs(65–69) and (70–74), (80-84)vs(65–69) and (70–74), (≥85)vs(65–69), (70–74) and (75-79); Female **p<0.001†**, (70-74)vs(65-69), (75-79) vs (65–69) and (70–74), (80-84)vs(65–69), (70–74) and (75-79), (≥85)vs(65–69), (70–74) and (75-79)<br>8UG: Male **p<0.001†**, (70-74)vs(65-69), (75-79)vs(65–69) and (70–74), (80-84)vs(65–69) and (70–74), (≥85)vs all AG; Female **p<0.001†**, (70-74)vs(65-69), (75-79)vs(65–69) and (70–74), (80-84) vs(65–69), (70–74) and (75-79), (≥85)vs AG<br>Body-mass index: Male p=0.123; Female **p<0.001†**, (≥85)vs (65–69) and (70–74)<br>Waist circumference: Male p=0.597; Female **p=0.001†** (80-84)vs (65–69) and (70–74)
**Nakamura et al., 2017** [64] (Japan)	Cross-sectional<br><br>n=142	74.26±5.86<br>[60, 90]<br><br>100%	*Objective measures:*<br>1. Cognitive function<br><br>2. Locomotive syndrome	1. Mini-Mental State Examination (MMSE)<br><br>2. 25-question Geriatric Locomotive Function Scale (GLFS-25).	Participants categorized into MMSE score ≤26 group and MMSE score >26 group, and into an GLFS-25 score ≤16 and GLFS-25 score >16.<br>Age was compared between the cognitive MMSE ≤26 and MMSE>26 groups and between the LS and non-LS groups by Student t-test.<br>The odds ratios (ORs) of measurements or LS status for an MMSE score ≤26 were calculated using univariate and multiple logistic regression analysis. Age, percent body fat, GLFS score, and LS were used as independent variables, and MMSE ≤26 as a dependent variable.	Student's t-test<br>MMSE >26 vs ≤26 groups **p=0.014†**<br>GLFS-25 >16 vs ≤16 groups **p<0.001†**<br><br>Univariate logistic regression analysis<br>Age OR=1.10, CI (1.02–1.20), **p=0.015†**

**Pisciottano et al., 2014** [68] (Brazil)	Cross-sectional<br>n=100	70.76±4.92<br>[65, 85]<br>100%	*Objective measures:*<br>1. Physical ability assessed by three performance tests<br>2. Appendicular lean mass (ALM) obtained by the sum of lean mass of arms and legs, total body fat (kg/ %)<br>3. Lower limbs strength assessed by peak torque for knee extension (KES) and flexion (KFS) at 60 ° per second	1a. Dynamic gait index (DGI)<br>1b. Timed Up and Go test (TUG)<br>1c. Berg Balance test (BBT)<br>2. Dual-emission Xray densitometer (DPX Md +, GE - Lunar Radiation Corporation, Madison, WI, Usa).<br>3. Isokinetic dynamometer (Cybex 125 dynamometer AP, Chattanooga, TN)	Correlations between age, anthropometric parameters, BMD, lean mass, fat mass, physical tests and strength were investigated by spearman and Pearson's correlation tests.<br>Variables with significant correlation were used for multiple linear regression models to identify the main outcomes in the study: ALM, KES, KFS and the physical tests (DGI, TUG and BBT)	Age correlations reported:<br>TUG, BBT and DGI r=0.28-0.14, **p<0.001†**; TUG r=0.28, **p<0.001†**; BTT, identified as significantly correlated but p-value non reported; KES r=-0.27, **p<0.001†**; KFS r=-0.22**, p<0.05†**<br>Multiple linear regression (Adjusted R^2 – R^2)<br>ALM-12.28+0.03xage+0.09xweight+11.38xheight+0.02xKES,R^2=0.72<br>KES 32.06-1.15xage+0.02xweight+52.17xheight+3.67xALM, R^2=0.35<br>KFS 21.85-0.78xage-0.05xweight+28.06xheight+2.65xALM, R^2=0.28<br>TUG 9.50+0.08xage+0.02xweight-4.08xheight-0.01xKES, R^2=0.18<br>BBT 59.13-0.11xage-0.03xweight+2.56xheight+0.01xKES, R^2=0.13<br>DGI 20.31-0.03xage-0.02xweight+2.38xheight+0.02xKES, R^2=0.10
**Chao et al., 2013** [41] (China)	Cross-sectional<br>n=849<br>AG<br>(60-64)<br>n=581<br>(65-79)<br>n=226<br>(80+)<br>n=38	67.4 ± 7.7<br>52.7%	*Self-reported measures:*<br>1. Body function, Self-care ability function, Emotional personality, Memory function and Social adaptability assessed by questionnaire	1. Elderly Health Assessment Scale	ANOVA was used to compare the Elderly Health Assessment subscales between different groups according to demographic classification [(60-64),(65-69),(70-74),(75-79), (80+)].<br>Ordinal logistic regression, was used to analyze the main factors affecting the Elderly Health Assessment Scale	ANOVA<br>Body Function F= 2.50, **p=0.04†**; Self-care ability F=6.49, **p<0.01†**<br>Physical health F=4.83, **p=0.01†**;Emotional personality F=1.03, p=0,41<br>Memory function F=7.65, **p<0.01†**; Mental health F=5.27, **p<0.01†**<br>Social adaptability F=2.36, p=0.05<br>Ordinal logistic regression<br>AG (60-80+) OR (95%CI)=2.01 (1.01-4.03), p=0,05<br>AG (70-80+) years OR (95%CI)=1.43 (0.84-1.84), p=0.30
**Ekström et al., 2016** [49] (Sweden)	Cross-sectional<br>n=768<br>AG<br>(67–70)<br>n = 371<br>(79–89)<br>n = 397	AG<br>md (q1-q3)<br>(67-70)<br>68 (67–69)<br>(79-89)<br>84 (81–87)<br>AG<br>(67-70)<br>57.1%<br>(79-89)<br>74.6%	*Self-reported measures:*<br>1. Environmental barriers and accessibility assessed by questionnaire<br>2. Perception physical environment support performance daily activities in the home assessed by questionnaire<br>3. Home tie assessed by questionnaire<br>4. External control beliefs in relation to home assessed by questionnaire<br>5. Housing satisfaction assessed with the single question<br>6. Psychological functio assessed by questionnaire<br>7. Symptoms assessed by checklist<br>8. Perceived functional independence (PFI) addressed by a single question<br>9. Total number of functional limitations calculated by the HE<br>10. ADL assessed by scale	1. Housing Enabler (HE) instrument<br>2. Usability in My Home questionnaire (UIMH)<br>3. 28-item Meaning of home questionnaire<br>4. Housing-related Control Beliefs Questionnaire (HCQ)<br>5. "Are you happy with the conditions of your home?" adapted from "Housing Options for Older People" Questionnaire<br>6. Ryff scales of Psychological Wellbeing<br>7. Presence of symptoms<br>8. "All in all, how would you evaluate your own independence, i.e. in performing activities of daily living?"<br>9. Presence of limitations scored between 0 to 12. Use of mobility devices<br>10. ADL Staircase	Differences between age cohorts were tested non-parametrically using the Mann–Whitney U test. P-values were corrected according to Bonferroni's method.	No. of environmental barriers (HE): Outdoors **p=0.004†**, Entrances **p=0.019†**, Indoors **p<0.001†**, Total **p<0.001†**<br>Accessibility score (HE): Outdoors **p<0.001†**, Entrances **p<0.001†**, Indoors **p<0.001†**, Total **p<0.001†**<br>UIMH: Activity p=0.289, Environmental p=0.777<br>Meaning of home: Physical p<0.001†, Behavioral p=1.0, Cognitive-emotional p=1.0, Social **p=0.027†**<br>Housing related control beliefs (HCQ) External control **p<0.001†**<br>Housing satisfaction p=1.0<br>Psychological well-being: Autonomy p=1.0, Purpose in life **p<0.001†**<br>Symptoms: Total p=0.126, Tension p=1.0, Gastro-urinary p=0.252, Musculoskeletal p=0.669, Metabolism p=0.201, Heart-lung p=1.0, Head **p<0.001†**<br>PFI **p<0.001†**<br>Functional limitations (HE) **p<0.001†**<br>Mobility device use<br>Indoors: Cane/crutch; Walking frame **p<0.001†**, Wheelchair p=1.0<br>Outdoors: Cane/crutch; Walking frame **p<0.001†**, Wheelchair p=0.028<br>Activities of daily living<br>Independent without difficulty **p<0.001†**; Independent with difficulty **p=0.036†**; Dependent IADL **p<0.001†**; Dependent IADL and PADL **p<0.001†**; Dependent IADL and PADL **p<0.001†**

**Fastame et al., 2020** [50] (Italy)	Cross-sectional*<br>n=53<br>AG<br>(80–85)<br>n=20<br>(90–95)<br>n=17<br>(99–105)<br>n=16	[80, 105]<br>AG<br>(80–85)<br>82.6±1.9<br>(90–95)<br>91.3±1.2<br>(99–105)<br>101.1±1.8<br>49,1%	*Self-reported measures:*<br>1. Perceived psychological well-being assessed by questionnaire<br>2. Socially desirable response assessed by inventory	1. Psychological Well-Being and Aging Questionnaire (PWAQ) [total score (PWAQ-tot), individual's coping strategies (PWAQ-COP), emotional competency (PWAQ-EC); personal satisfaction (PWAQ-PS)]<br>2. Marlowe and Crown Social Desirability Scale (MCSDS)	The effect of age group on total and subscales of psychological well-being scores was determined using Multivariate Analysis of Covariance (MANCOVA). Social desirability was included as a covariate.<br>ANOVA was conducted to explore the impact of age group on the self-reported social desirability dimension.	Psychological well-being Wilks' Lambda=0.62, df=8;92, **p=0.004†**<br>Social desirability Wilks' Lambda=0.83, df=4;46, p=0.07<br>PWAQ-COP dimension F(2,49)=6.72, **p=0.003†**; PWAQ-tot F(2,49)=0.97, p=0.39; PWAQ-EC F(2,49)=0.50, p=0.61; PWAQ-PS F(2,49)=1.06, p=0.35<br>Bonferroni post-hoc comparisons:<br>PWAQ-COP (99–105) vs (80–85) **p=0.002†**; (99–105) vs (90–95) p=0.14 ; (80–85) vs (90–95) NR<br>ANOVA: Social desirability F(2,50) = 2.43, p=0.10
**Hara et al., 2020** [54] (Japan)	Cross-sectional*<br>n=74	76.8 ± 9.0<br>Male<br>md (IR)<br>77 (71.5-82.5)<br>Female<br>77 (74-82)<br>68.9%	*Objective measures:*<br>1. Number of teeth assessed by dentist<br>2. Masticatory function assessed by maximum bite force (MBF)<br>3. Muscle quantity assesssed by masseter muscle thickness (MMT)<br>4. Muscle quality assessed by masseter muscle echo intensity (MMEI)<br>5. Movement during contraction assessed by the displacement of the masseter muscle during forceful biting (MMD) and time of force production (TFP)	1. NA<br>2 .Pressure-sensitive film (Dental prescale 50 HR Type R; Fujifilm) and OCCLUZER device (FPD-707; Fujifilm).<br>3. 4. Ultrasonic diagnostic apparatus (MySono U6; Samsung Medison, Inc, Seoul, Korea) with a linear probe (broadband frequency, 5-12 Hz; gain, 45 dB; depth, 2.7 cm)<br>5. M-mode ultrasonography with a linear probe	Correlations objective measures, and age were assessed using bivariate correlation analysis, using Pearson's correlation analysis for parametric data and Spearman's rank test for non-parametric data.<br>Correlations among MBF, MMEI and MMT, was tested by multivariate linear regression analysis for MBF, with MMT and MMEI (model 1); thereafter, MMD and TFP were added (model 2).<br>To study the relationship among MMD, MMT and MMEI, multivariate linear regression analysis for MMD was performed, with adjustment for number of teeth, age and sex.	Correlations<br>Number of teeth r=-0.505, **p<0.05†**; MBF r=-0.228, **p=0.049†**<br>MMT r=-0.273, **p<0.05†**; MMEI r=0.112, p>0.05; MMD r=0.153, p>0.05; Time of force production r=0.023, p>0.05<br>Multivariate linear regression analysis for MBF<br>Model 1 (Age, Sex, Number of teeth, MMT, MMEI) R^2=0.386<br>Age B=11.192, 95% CI (-5.707, 28.090), β=0.157, p=0.191, Variance Inflation Factor VIF=1.591<br>Model 2 (Age, Sex, Number of teeth, MMT, MMEI, MMD, TFP) R^2=0.440<br>Age B=6.532, 95% CI (-10.373, 23.437), β=0.092, p=0.443, VIF=1.666<br>Multivariate linear regression analysis for MMD R^2=0.417<br>Age B=0.034, 95% CI(-0.005, 0.074), β=0.204, p=0.088, VIF=1.594
**Hayashi et al., 2002** [55] (Japan)	Cross-sectional<br>n=151	70 ± 5<br>[60, 80]<br>100%	*Objective measures:*<br>1. Physical strength assessed by grip strength, vertical jump height, jumping reaction time, time required to get up from a recumbent position to sitting down on a chair, sit and reach distance, time of balancing on one leg with eyes closed and tapping score for 30 seconds<br>2. Cerebral functions measuring occupational aptitude by questionnaire<br>*Self-reported measures:*<br>3. Mental health status assessed by screening questionnaire	1. Digital grip dynamometer (Takei Kiki Co., Tokyo, Japan), Jump-meter (VINE Co., Tokyo, Japan), tapping counter (Takei Kiki Co., Tokyo, Japan)<br>2. General Aptitude Test Battery (GATB)<br>3. General Health Questionnaire (GHQ)	Bivariate correlation coefficients (Spearman's rank correlation coefficients) between age and physical strength, cerebral functions, and GHQ scores	Physical strengths<br>Grip strength (kg) r=-0.240, **p<0.01†**; Vertical jump height (cm) r=-0.301, **p<0.001†**; Jumping reaction time (sec) r=0.216, **p<0.01†**; Time to stand up and sit down (sec) r=0.204, **p<0.05†**; Sit and reach distance (cm) r=-0.197, **p<0.05†**; Time of balancing on one leg with eyes closed r=-0.343, **p<0.001†;** Tapping score (beat/sec) r=-0.251, **p<0.01†**<br>General Aptitude Test Battery sub-tests<br>Motor coordination r=-0.411, **p<0.001†**; Form perception r=-0.190, **p<0.05†**; Verbal aptitude r=-0.213, **p<0.01†**; Spatial perception r=-0.147, p>0.05; Manual dexterity r=-0.369, **p<0.001†**; Finger dexterity r=-0.425, **p<0.001†**<br>GHQ sub-scales<br>Somatic symptoms r=-0.021, p>0.05; Anxiety/insomnia r=-0.051, p>0.05; Social dysfunction r=0.162, **p<0.05†**; Severe depression r=0.224, **p<0.01†**

**Neri et al., 2012** [65]<br>(Brazil)	Cross-sectional*<br><br>n=900	72.78±5.82<br>[65-97]<br><br>Performed MMSE sentence<br>72.24±5.74<br>Not performed MMSE sentence<br>73.74±5.85<br><br>69.3%	*Objective measures:*<br>1. Cognitive status<br><br>2. Language performance	1. MMSE<br><br>2. MMSE sentence (Verbal fluency - number of words produced; Grammatical complexity - number of phrases or interrelated ideas)	Chi-square test used to evaluate the performance in the MMSE sentence, stratified by age (65–69; 70–74; 75–79; ≥80)<br><br>Participants who responded to the MMSE sentence was compared to those who did not answer as to age (65–69; 70–74; 75–79; ≥80) by Mann–Whitney U test.<br><br>Comparison of cognitive and textual performance according to age Kruskal–Wallis test and Dunn post hoc	Performance in the MMSE sentence by age group<br>$\chi2$ =24.18, df=9, **p=0.004†**<br><br>Age comparison of elderly adults whose answers to the MMSE sentence were included versus excluded<br>**p<0.001†**<br><br>Comparison of cognitive and textual performance according to age<br>MMSE Total Score **p<0.001†**, (≥80) vs (65-69)<br>Number of Words p=0.101<br>Number of ideas p=0.681
**Salvà et al., 2005** [72]<br>(Spain)	Cross-sectional<br><br>n=443	74,6 ± 6.9<br><br>59,1%	*Objective measure:*<br>1. Gait and Balance assessed by test	1. Performance Oriented Mobility Assessment (POMA)	Comparison of POMA impairments between age groups (< 71, 71-75, 76-80, 81-85, > 85) by Kruskal-Wallis test and Mann-Whitney test to compare impairments between two groups	Kruskal-Wallis test<br>3 or more impairments in POMA **p<0.001†**<br><br>Mann-Whitney test<br>< 71, 71-75 vs >85, **p<0.001†**
**Choudhary, 2020** [43]<br>(India)	Cross-sectional*<br><br>n=60<br>AG<br>(60-69)<br>n=20<br>(70-79)<br>n=20<br>(80-89)<br>n=20	(60-69)<br>63.95±2.50<br><br>(70-79)<br>73.35±2.36<br><br>(80-89)<br>83.35±2.25<br><br>50%	*Objective measures:*<br>1. Functional performance assessed by number of step performed in 6 minutes<br><br>2. Balance assessed by the time to complete sequence of directions change	1. 6 minute step test (6MST)<br><br>2. Four Square Step	ANOVA was used to study the difference among scores of Six Minute Step Test and Four Square Step test for subjects in all the 3 age groups.	Six Minute Step Test<br>Significant difference between<br>(60-69) vs(70-79); (70-79) vs (80-89); (80-89) vs (60-69)<br>F-value=21.96, **p=0.000†**<br><br>Four Square Step Test<br>Significant difference between<br>(60-69) vs (70-79); (70-79) vs (80-89); (60-69) vs (80-89)<br>F- value=8.97, **p=0.000†**
**Confortin & Barbosa, 2015** [45]<br>(Brazil)	Cross-sectional<br><br>n=270	73.2 ± 8.8<br>[60, 100]<br><br>100%	*Objective measures:*<br>1. Lower limb muscle strength/resistance (LLMS) assessed by time to stand up and sit down 5 times<br><br>2. Handgrip strength (HGS) assessed by the maximum of two grip pressure trials	1. “Chair stand” test<br><br>2. Mechanical dynamometer<br>(Takei Kiki Kogyio TK 1201, Japan)	Poisson regression (crude and adjusted; 95%CI) to verify association between HGS, LLMS and age. The variables that presented statistical significance of at least 20% (p≤0.20) in the crude analyses were included in the adjusted analyses, according to the hierarchical model. At each level of the adjusted analyses, the backward selection technique was used. Level 1, 2 and 3 variables were adjusted among themselves, and those that obtained p≤0.20 were kept in models for adjustment in the following levels.	LLMS<br>Crude Analysis (Test of Wald for linear trend)<br>PR (95% CI) 1.04 (1.03-1.06), **p≤0.001†**<br>Adjusted Analysis (Test of Wald for linear trend)<br>1.03 (1.02-1.05), **p≤ 0.001†**<br><br>HGS<br>Crude Analysis (Test of Wald for linear trend)<br>PR prevalence ratio (95% CI) 1.03 (1.01-1.05), **p=0.006†**<br>Adjusted Analysis (Test of Wald for linear trend)<br>1.03 (1.01-1.05), **p=0.02†**

**Incel et al, 2009** [58] (Turkey)	Cross-sectional<br>n=24<br>AG<br>Younger elderly (65–70)<br>n=13<br>Older elderly (70–79)<br>n=11	69.16±3.73 [65, 79]<br>AG<br>(65–70)<br>66.46±1.45<br>(70-79)<br>72.18±2.81<br>62.5%	*Objective measures:*<br>1. Handgrip strength assessed by dinamometry<br>2. Tip pinch strength assessed by finger prehension force<br>*Self-reported measures:*<br>3. Self-estimated hand function (SEHF) assessed by scale<br>4. Disability assessed by a scale<br>5. IADL assessed by a scale<br>6. General health assessed by scale	1. Jamar dynamometer (JA Preston Corp., Ontario, Canada)<br>2. Manual pinchmeter that measures finger prehension force<br>3. Visual analog scale for the perception of the use the hand to perform activities<br>4. Duruo¨ z hand index (Cochin scale)<br>5. IADL scale (Lawton and Brody, 1969)<br>6. Short form 36	Comparisons between older adults subgroups (according to sex and age) were tested using t-tests and Mann–Whitney U test.	Handgrip strength p>0.05<br>Tip pinch strength p>0.05<br>SEHF p>0.05<br>Dreiser hand function index p>0.05<br>IADL p>0.05<br>SF-36 p>0.05
**Sauvel et al, 1994** [74] (France)	Longitudinal Study*<br>(1 year)<br>n=1850	NR<br>NR	*Self-reported measures:*<br>1. ADL dependence assessed by index<br>2. IADL dependence assessed by questionnaire<br>3. Mobility assessed by scale	1. Katz Index of ADL<br>2. Instrumental activities of daily living by Lawton & Brody, 1969<br>3. Confinement scale	Age differences between individuals that developed dependence after one year and individuals who maintained independence in ADL, IADL and Mobility assessed by Chi-squared test.<br>Independent effect of age in becoming dependent assessed by logistic regression for ADL, IADL and Mobility	Chi-squared test<br>ADL **p<0.001†**<br>IADL **p<0.001†**<br>Mobility **p<0.001†**<br>Logistic regression<br>ADL OR=1.07, CI 95%=1.04-1.11<br>IADL OR=1.11, CI95%=1.07-1.14<br>Mobility OR=1.09, CI95%=1.05-1.14
**Sherman & Reuben, 1998** [75] (USA)	Cross-sectional<br>n=363	75.9 ± 5.9<br>NR	*Objective measures:*<br>1. Lower and upper extremity function<br>2. Lower extremity function, balance gait, strength and endurance assessed by test	1. Physical Performance Test<br>2. National Institute on Aging Battery	Comparison of the age between the group below the median and above the median of Physical Performance Test and National Institute on Aging Battery (test non reported)	Physical Performance Test<br>Age **p<0.0001†**<br>National Institute on Aging Battery<br>Age **p<0.0001†**

**Smee et al., 2012** [76]<br>(Australia)	Cross-sectional*<br><br>n=32<br><br>AG<br>65–74<br>n = 10<br>75–84<br>n = 16<br>>85<br>n = 6	77.9 ± 7.7<br>[65-92]<br><br>AG<br>65-74<br>68.8 ± 2.7<br>75-84<br>79.6 ± 3.26<br>(>85)<br>88.8 ± 7.70<br><br>47%	*Subjective measures:*<br>1. General health assessed by scale<br><br>*Objective measures:*<br>2. Physical functionality assessed tests	1. 12-Item Short-Form Health Survey (SF-12) with the physical (physSF-12) and mental (mentSF-12) subscales identified<br><br>2. Continuous-Scale Physical Functional Performance (CS-PFP 10) (total score and five physical domains—upper body strength, lower body strength, upper body flexibility, balance, and endurance)	Test comparing General health and Physical functionality between age groups are non-reported.<br><br>Pearson product-moment correlation coefficients were used to examine bivariate relationships between all variables and age	CS-PFP10 total score (65-74) vs (>85), **p<0.01†**<br>Upper body strength (65-74) vs (>85), **p<0.01†**<br>Lower Body strength (65-74) vs (>85), **p<0.01†**<br>Upper body flexibility NS<br>Balance (65-74) vs (>85), **p<0.01†**<br>Endurance (65-74) vs (>85), **p<0.01†**<br>SF12: physSF12 NS; mentSF12 NS<br>Pearson Correlation<br>CS-PFP10 total score r=−0.61, **p<0.01†**<br>Upper body strength r=−0.45, **p<0.01†**<br>Lower Body strength r=−0.52, **p<0.01†**<br>Upper body flexibility r=−0.44, **p<0.05†**<br>Balance r=−0.63, **p<0.01†**<br>Endurance r=−0.62, **p<0.01†**<br>SF12phys r=−0.28, p>0.05; SF12men r=−0.04, p>0.05
