# Supplementary material for: Indicators and Instruments to Assess Components of Disability in Community-Dwelling Older Adults: A Systematic Review
Source: Sensors (Basel). 2022 Oct 28;22(21):8270. doi: 10.3390/s22218270 (PMC9653663; doi:10.3390/s22218270)
Supplement: Supplementary file 1 [file sensors-22-08270-s001.zip › Table_S3.pdf]

Table S3. Characterization and summary of studies included in the review with classification of "Poor" in Downs and Black scale, listed by decreasing order of quality score (for those with same score alphabetic order was used).

Notes: AG – age group, ANOVA - Analysis of variance; M±SD – Mean plus Standard Deviation; NR – non reported; NS – non significant, * - study design non reported in the respective study, and thus classified by the reviewers; † - indicators with significative change or association with age.

| ID | **Study Design** (Follow-up time) **Sample Size** | **Age** M±SD [range] % female | **Indicators** Objective & Self-reported | **Instrument** | **Main statistical strategy used to assess the influence of age** | **Significative change/ association with age** |
|---|---|---|---|---|---|---|
| **Andrade et al., 2018** [36] (Brazil) | Cross-sectional* <br> n=81 <br> AG <br> (60-69) <br> n=30 <br> (70-79) <br> n=40 <br> (≥80) <br> n=11 | NR <br> 58% | *Objective measures:* <br> 1. Lower extremities strength/endurance assessed by time to perform 5 stand-to-sit <br> 2. Postural control parameters assessed by the recording of co-ordinates of the body's CoP during 30 seconds | 1. Chair stand test <br> 2. Piezoelectric force platform (Footwork Pro AM CUBE, France) | Kurskall-Wallis test was used to compare lower limb strength and stabilometric variables among the studied age groups and a Mann-Whitney test with Bonferroni correction for multiple comparisons was used as a post-hoc test | Chair stand test <br> (≥80) - (60-69), **p<0.05†** <br> Co-ordinates of the body's CoP <br> Normalized AnteroPosterior displacement <br> (≥80) - (60-69) **p<0.05†** <br> (≥80) - (70-79) **p<0.05†** <br> Normalized MedioLateral displacement <br> (70-79) - (60-69) **p<0.05†** <br> (≥80) - (60-69) **p<0.05†** |
| **Poon et al., 1992** [69] (USA) | Cross-sectional* <br> n=165 | ≥60 <br> NR | *Objective measures:* <br> 1. Fluid and crystallized intelligence estimated by subtests of vocabulary, arithmetic, picture arrangement and block design <br> 2. Acquisition (primary memory) and retrieval (secondary memory) of information evaluated by a task <br> 3. Tertiary memory measured by a test <br> 4. Problem-solving ability evaluated by problem resolution | 1. Wechsler Adult Intelligence Test <br> 2. Learning task in which pairs of high and low frequency words were employed <br> 3. Test of the names and faces of six immediate past presidents of the United States <br> 4. Everyday problems encountered by most people living at home | Influences of Age Group on Cognitive Performances by ANOVA (age group 60s, 80s, ≥100) | Influences of Age Group on Cognitive Performances <br> Fluid intelligence F=33.85 (2,137), **p=0.0001†** <br> Crystallized intelligence F=25.72 (2,149), **p=0.0001†** <br> Primary memory F=41.73 (2,149), **p=0.0001†** <br> Secondary memory F=33.79 (2,146), **p=0.0001†** <br> Tertiary memory F=48.39 (2,160), **p=0.0001†** <br> Problem solving F=2.22 (2,161) p=0.1123 |
| **Araújo & Ribeiro., 2011** [37] (Portugal) | Cross-sectional* <br> n=991 <br> AG <br> (65-69) <br> n=290 <br> (70-79) <br> n=494 <br> (≥80) <br> n=207 | 74,1 ± 6.5 <br> [65, 101] <br> 70,4% | *Self-reported measures:* <br> 1. Subjective Health assessed trough 3 questions <br> 2. Objective Heath assessed trough 3 questions <br> 3. Function assessed by questionnaire | 1. General health self-assessment -"In general, do you think your health is…?"; Self-assessment of health compared to the past (question NR); Self-assessment of health compared to others "Comparing with those close to you, do you think your health is…?" <br> 2.1 Medical diagnoses (presence of diseases for which participants took medication) <br> 2.2 Vision ability <br> 2.3 Audition ability <br> 3. Scale of ADL | Descriptive analysis of selected variables, controlling for different sociodemographic variables, in three age groups: (65-69), (70-79) and (≥80). Statistical tests non reported. | Subjective Health <br> General health self-assessment p= 0.269 <br> Self-assessment of health compared to the past p=0.052 <br> Self-assessment of health compared to others **p<0,05†** <br> Objective Heath <br> Medical diagnoses p=0,08 <br> Vision ability **p< 0,05†** <br> Audition ability **p< 0,05†** <br> Function <br> ADL **p<0,05†** <br> IADL **p<0,05†** |
